# (−)-Epigallocatechin Gallate Targets Notch to Attenuate the Inflammatory Response in the Immediate Early Stage in Human Macrophages

**DOI:** 10.3389/fimmu.2017.00433

**Published:** 2017-04-10

**Authors:** Tengfei Wang, Zemin Xiang, Ya Wang, Xi Li, Chongye Fang, Shuang Song, Chunlei Li, Haishuang Yu, Han Wang, Liang Yan, Shumei Hao, Xuanjun Wang, Jun Sheng

**Affiliations:** ^1^College of Life Science, Jilin University, Changchun, China; ^2^Key Laboratory of Pu-er Tea Science, Ministry of Education, Yunnan Agricultural University, Kunming, China; ^3^State Key Laboratory for Conservation and Utilization of Bio-Resources in Yunnan, Kunming, China; ^4^Pu’er Institute of Pu-erh Tea, Pu’er, Yunnan, China

**Keywords:** (−)-epigallocatechin gallate, Notch, macrophages, laminin receptor, inflammation

## Abstract

Inflammation plays important roles at different stages of diabetes mellitus, tumorigenesis, and cardiovascular diseases. (−)-Epigallocatechin gallate (EGCG) can attenuate inflammatory responses effectively. However, the immediate early mechanism of EGCG in inflammation remains unclear. Here, we showed that EGCG attenuated the inflammatory response in the immediate early stage of EGCG treatment by shutting off Notch signaling and that the effect did not involve the 67-kDa laminin receptor, the common receptor for EGCG. EGCG eliminated mature Notch from the cell membrane and the nuclear Notch intercellular domain, the active form of Notch, within 2 min by rapid degradation *via* the proteasome pathway. Transcription of the Notch target gene was downregulated simultaneously. Knockdown of Notch 1/2 expression by RNA interference impaired the downregulation of the inflammatory response elicited by EGCG. Further study showed that EGCG inhibited lipopolysaccharide-induced inflammation and turned off Notch signaling in human primary macrophages. Taken together, our results show that EGCG targets Notch to regulate the inflammatory response in the immediate early stage.

## Introduction

The inflammatory response has an important role at different stages of diabetes mellitus (DM), tumorigenesis, and cardiovascular diseases (1, 2). The inflammatory response is common in the intestine and gut due to microbe invasion and damage to cells in epithelial barriers (3). Attenuation of the inflammatory response has been considered an important side effect in the therapy and prevention of cancer, DM, cardiovascular diseases, and Alzheimer’s disease (4, 5). Compounds in food and drinks that can help to attenuate inflammation have been studied for their effects and mechanisms of action (6, 7).

(−)-Epigallocatechin gallate (EGCG) is found in Chinese green tea and Pu’er tea. It has been proposed to have strong anti-inflammatory effects (8, 9). The mechanisms of EGCG in the attenuation of inflammation have been explored. EGCG downregulates the phosphorylation of nuclear factor-kappa B (NF-κB), a key regulator of the classical pathway of the inflammation response induced by lipopolysaccharide (LPS) (10). In addition, the 67-kDa laminin receptor (67LR), which has been proposed to be a receptor for EGCG on cell membranes, has been shown to downregulate the anti-inflammatory effects of EGCG (11). However, those studies have focused on the physiologic response of cells ≥6 h after EGCG treatment. Thus, those results may indicate that EGCG treatment interferes with mediator proteins in the signal transduction pathway, although the original mechanism of EGCG treatment is not clear. Such cells have undergone several rounds of signaling processes in ≥6 h, and cytokine release and feedback may have already occurred in those cells. To understand the detailed mechanisms of EGCG in inflammation, we examined the immediate early response of macrophages after EGCG addition and the underlying mechanisms.

The mechanisms of the inflammatory response have been studied extensively. The NF-κB molecule has been considered to be a key regulator/mediator of LPS-induced inflammation (12). The well-known mitogen-activated protein kinase (MAPK) pathway, which plays a key part in many other biologic processes, has also been confirmed to participate in the inflammatory response (13).

Notch signaling has a role in the development of multicellular organisms (14). Notch has been shown to function during development of the immune system, including the maturation of T cells (15). In recent years, the Notch signaling pathway has been found to have an essential role in regulation of the inflammatory response (16). Notch and toll-like receptor (TLR) pathways cooperate to activate canonical Notch target genes, including the transcriptional repressors hairy and enhancer of split-1 (Hes1) and hairy/enhancer of split related with YRPW motif protein 1 (Hey1), and to increase the production of the canonical TLR-induced cytokines, tumor necrosis factor (TNF), interleukin (IL)-6, and IL-12 (17–19). Cooperation of these pathways to increase expression of target genes is mediated by the Notch pathway component and transcription factor recombination signal-binding protein for immunoglobulin (Ig) kappa J region (17). Basal activation of the Notch signal is required to amplify the signal transduction from TLRs. The Jagged-1/Notch signaling pathway is considered a potential molecular mechanism underlying the EGCG attenuation of oxidized low-density lipoprotein–induced dysfunction of the vascular endothelium (20). Studies have shown that EGCG may prevent the uric acid-induced inflammatory effect in human umbilical vein endothelial cells in relation to Notch1 (21). The Notch pathway has also been proposed to be related to the effects of EGCG in the protection of cochlear hair cells from ototoxicity and inhibition of the proliferation of colorectal cancer cells (22, 23). These observations suggest a potential relationship between EGCG and the Notch pathway.

Here, we studied the immediate early mechanism of EGCG on the inflammatory response induced by LPS in human macrophages derived from THP-1 cells. We found that EGCG did not alter the phosphorylation levels of NF-κB or the phosphorylation state of the key MAPK molecules p38, extracellular signal–regulated kinase (ERK), and c-Jun-*N*-terminal kinase (JNK) during the first hour after EGCG treatment. However, EGCG suppressed Notch signaling, which is necessary for an increased inflammatory response. Knockdown of Notch1/2 expression impaired the effects of EGCG upon inflammation.

## Materials and Methods

### Cell Culture and Transfection

The human monocyte cell line (THP-1) was purchased from the Cell Bank in the Chinese Academy of Sciences (Kunming). Cell lines were cultured in Roswell Park Memorial Institute (RPMI) 1640 medium supplemented with 10% (*v/v*) fetal bovine serum (Biological Industries, USA) and 50 μM β-mercaptoethanol. Infection was carried out using Notch 1 or Notch 2 short hairpin RNA (shRNA) Lentiviral Particles (Santa Cruz Biotechnologies, USA) according to manufacturer instructions.

### Macrophage Differentiation

THP-1-derived macrophages were differentiated from THP-1 by propidium monoazide (PMA) treatment (24). THP-1 cells were counted to a density of 5 × 10^5^ cell/mL, and viability was >95% as determined by exclusion of Trypan blue dye. Then cells were seeded on 60-mm culture dishes (5 × 10^6^ per dish) in RPMI 1640 supplemented with 10% fetal bovine serum (Biological Industries) and treated with PMA (10 ng/mL) for 48 h. This was followed by incubation with serum-free buffer for a further 24 h at 37°C in a humidified incubator in an atmosphere of 5% CO_2_. THP-1-derived macrophages were washed twice with warm RPMI 1640 to remove non-adherent cells.

### Isolation of Peripheral Blood Mononuclear Cells (PBMCs) and Macrophage Differentiation

Peripheral blood mononuclear cell-derived macrophages were obtained from anticoagulated, pathogen-free human peripheral blood using Medium for Human Lymphocyte Separation (Solarbio, China) in accordance with the manufacturer instructions. The upper layer was retained as autologous serum, which was used to culture PBMC-derived macrophages. The mononuclear cells from PBMCs were washed twice with serum-free RPMI 1640 and resuspended in RBPI 1640 with 5% autologous serum. Then cells were cultured for 7 days with 50 ng/mL macrophage colony-stimulating factor-1 in a humidified incubator (37°C, 5% CO_2_).

### 67LR Blockade

THP-1-derived macrophages were incubated with RPMI 1640 containing 5 μg/mL anti-67LR MLuC5 antibody (Santa Cruz Biotechnology) or isotype-matched control mouse IgM (Santa Cruz Biotechnology) at 37°C in a humidified incubator containing 5% CO_2_ for 1 h before the addition of EGCG or LPS.

### Assay for Inflammatory Cytokines

The concentrations of TNF-α and IL1-β were determined using enzyme-linked immunosorbent assay (ELISA) kits (Dakewe, China) according to manufacturer instructions. Inflammatory cytokines released in the medium were monitored by Inflammation Antibody Array 3 (Norcross, USA) according to manufacturer instructions. THP-1-derived macrophages were pretreated with EGCG (50 μg/mL) for 30 min and stimulated with LPS (200 EU/mL) for a further 3 h. PBMC-derived macrophages were pretreated with EGCG (50 μg/mL) for 30 min and stimulated with LPS (200 EU/mL) for a further 3 or 6 h. Data were collected using a chemiluminescence imaging system (Fluor Chem E; ProteinSimple, USA) and analyzed using the RayBio^®^ Human Inflammation Antibody Array 3 Analysis Tool.

### Reverse Transcription Polymerase Chain Reaction (RT-PCR) and Quantitative RT-PCR

THP-1-derived macrophages were pretreated with EGCG (50 μg/mL) for 30 min and stimulated with LPS (200 EU/mL) for a further 1 h. RNA was isolated using TransZol^®^ (Transgen, China). Reverse transcription of RNA was done using a PrimeScript™ RT Reagent kit with gDNA Eraser (TaKaRa Bio, Japan). Quantitative PCR was carried out on a 7900HT Real-Time PCR system (Applied Biosystems, USA) using primers (Table S1 in Supplementary Material) for human *actin, il1 beta, il6, il8, il10, mcp1, ccl3, ccl4, ccl5, tnf alpha, timp2, bhle40, ddit4, hk2, p4ha1, pfkfb3, rhou, elmo1, hes1*, and *hey1* using SYBR Green PCR Master Mix (TaKaRa Bio). All reactions were performed in triplicate and normalized to actin expression.

### Immunoprecipitation and Posttranslation Modification Assay

For immunoprecipitation, cells were treated with indicated reagents before lysis in lysis buffer (50 mM Tris-HCl, pH 8.0; 120 mM NaCl; 0.5% NP-40) supplemented with a protease inhibitor cocktail (Sigma–Aldrich, USA) and phosphatase inhibitor cocktail (Sigma–Aldrich). After preclearing with protein A/G resin, lysates were incubated with Notch2 antibody for 24 h at 4°C with gentle rotation. Protein A/G resin was added to lysates and incubated for a further 6 h. Then protein A/G resin was spun down and washed five times with lysis buffer, and Notch2 was eluted with sodium citrate (pH 2.5). Notch2 eluates were neutralized with 1.5 M Tris–Cl (pH 8.8) and incubated at 37°C with ataxin-3 and peptide-*N*-glycosidase F (PNGase F) separately. The digested Notch2 was analyzed with a western blotting probe with the indicated antibodies.

### Western Blotting

Cells were lysed in 150 μL lysis solution (Beyotime, China) containing phenylmethylsulfonyl fluoride (1 mM). Whole-cell lysate samples were separated by 8% sodium dodecyl sulfate–polyacrylamide gel electrophoresis and transferred to polyvinylidene difluoride membranes (Millipore, USA). The latter were blocked in 5% bovine serum albumin and incubated with primary antibody overnight at 4°C and incubated with horseradish peroxidase-conjugated secondary antibody for 1 h at room temperature. Antibodies against phospho-NF-κB, phospho-Erk1/2, phospho-p38 MAPK, Hes1, Actin, Notch1, and Notch2 were purchased from Cell Signaling Technology (USA). Anti-rabbit IgG and anti-rabbit IgG were purchased from R&D Systems (USA).

### Notch Reporter Assays

A total of 293 cells were cotransfected transiently with 16 Notch-responsive element CSL (CBF1, Suppressor of Hairless, Lag-1, CSL)/mini-promoter firefly luciferase reporter (constructed by our research team) and constitutively expressed *Renilla* luciferase reporter (pRL-CMV; Promega, USA). EGCG was added alone or together with ligand-expressing cells (HepG2 cells) 18 h after transfection. Luciferase activities were measured 12–24 h after EGCG addition (Dual Glo Luciferase; Promega). Typically, three replicates were analyzed for each condition, and values were expressed as relative luciferase units (firefly signal divided by the *Renilla* signal).

### Molecular Interaction Assay

To measure a possible direct interaction between Notch and EGCG, we prepared the human Notch negative regulation region (NRR). The NRR has been considered to have a key role in Notch activation. The binding affinity of EGCG and NRR was determined using an Octet Red96 system (ForteBio, USA). The biotinylated NRR protein was loaded on Super Streptavidin biosensors and acted with gradient concentration of EGCG in the assay buffer (phosphate-buffered saline, pH 6.5). We measured EGCG association and dissociation for 60 s each. Kinetic parameters and affinities were calculated from a non-linear global fit of the data between EGCG and NRR using Octet Data Analysis v7.0 (ForteBio).

### Statistical Analyses

Data are represented as mean ± SD. Data were analyzed using Student’s *t*-test with GraphPad (USA). *p < 0.05, **p < 0.01, and ***p < 0.001 were considered significant.

## Results

### EGCG Attenuated the Immediate Early Inflammatory Response

To evaluate the direct effects of EGCG on inflammation, human macrophages differentiated from THP-1 cells (Figures S1 and S2 in Supplementary Material) for 48 h with PMA (10 ng/mL) were pretreated with EGCG (50 μg/mL) for 30 min and then stimulated with LPS (200 EU/mL) for an additional 6 h. Samples of the medium were collected 3 h after LPS stimulation. The concentration of major inflammatory cytokines was measured using ELISA kits.

**Figure 1:**
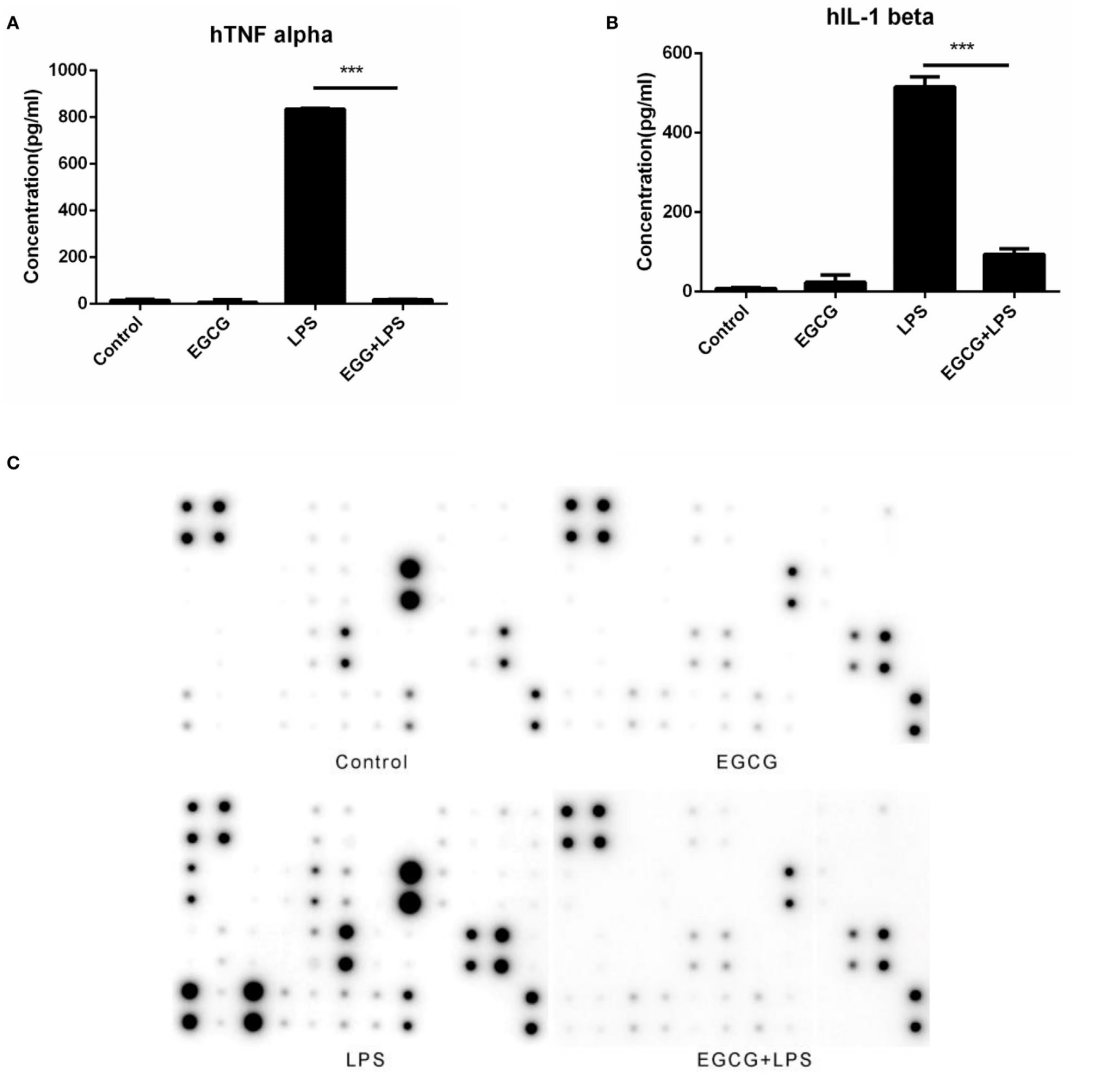

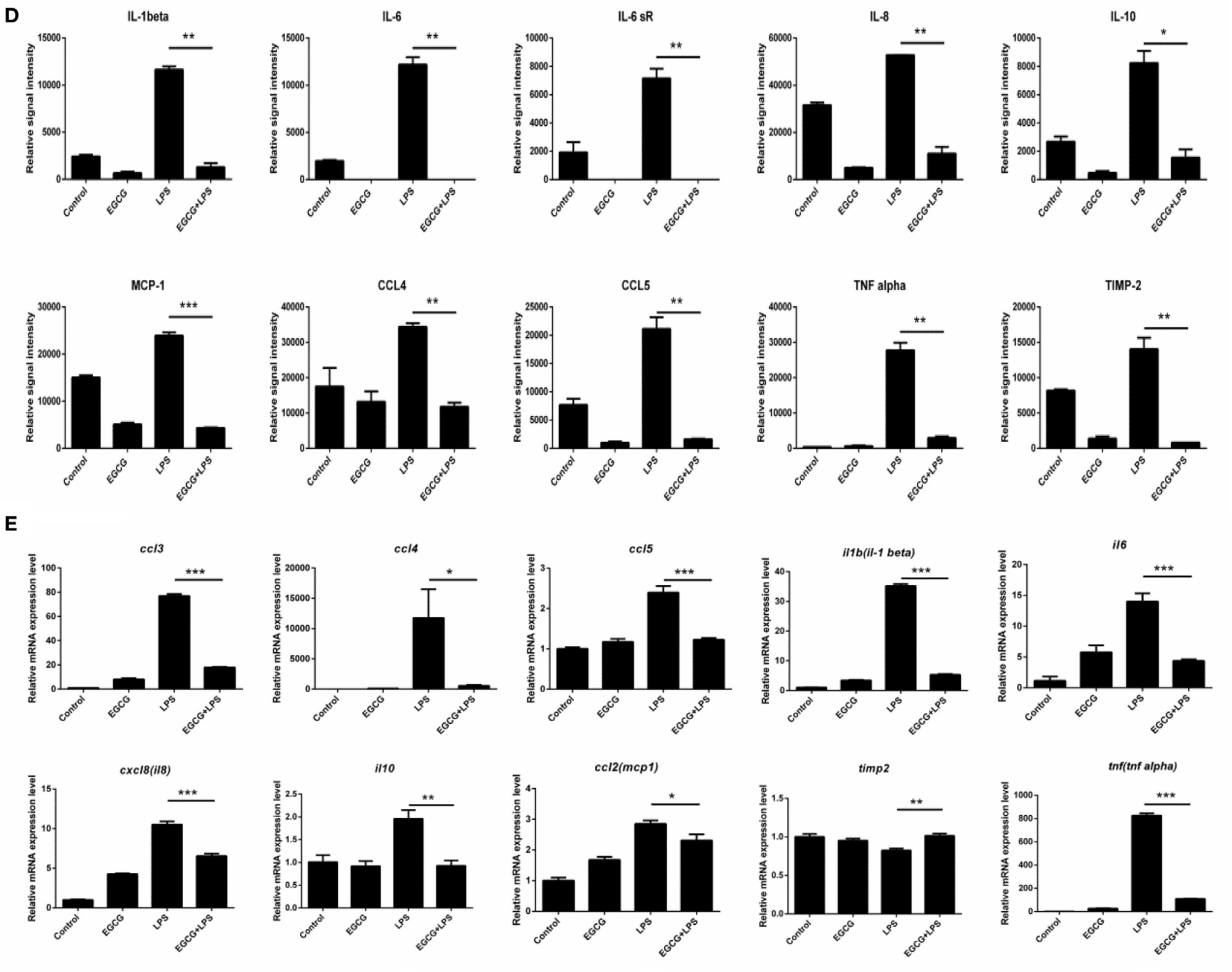
**(−)-Epigallocatechin gallate (EGCG) attenuated the inflammatory response in the immediate early stage**. THP-1-derived macrophages were pretreated with EGCG (50 μg/mL) for 30 min before exposure to lipopolysaccharide (LPS) (200 EU/mL) for 3 h. Expression of the inflammatory cytokines in the culture medium was measured by enzyme-linked immunosorbent assay **(A,B)** and a RayBio^®^ C-Series human inflammation antibody array **(C)**, and chips were scanned and analyzed **(D)**. mRNA levels 1 h after LPS treatment were measured by real-time polymerase chain reaction **(E)**. Data are represented as mean ± SD (*n* = 3). Differences between the two groups were assessed by two-way ANOVA using GraphPad. Representatives of three independent experiments with similar results are shown. Results are represented as mean ± SD (*p < 0.05, **p < 0.01, and ***p < 0.001).

Lipopolysaccharide stimulated the release of TNF-α (Figure 1A) and IL-1β (Figure 1B) significantly (*p* < 0.001), and EGCG attenuated release of these cytokines (Figures 1A,B) in human macrophages. To determine the effects of EGCG on inflammation in macrophages, we examined expression of inflammatory cytokines in human macrophages in culture supernatants collected 3 h post-LPS treatment under identical experimental conditions using a human inflammation antibody array (RayBio). Expression of all inflammatory cytokines was increased significantly (*p* < 0.05, *p* < 0.01, or *p* < 0.001) by LPS stimulation, as well as by TNF-α and IL-1β (Figures 1C,D). Expression of 24 of 40 inflammatory factors induced by LPS was reduced significantly (*p* < 0.05, *p* < 0.01, or *p* < 0.001) by EGCG, including IL-1β, IL-6, IL-8, IL-10, monocyte chemotactic protein 1 (MCP-1), macrophage inflammatory protein 1-alpha (MIP-1α), MIP-1β, IL-12-p70, regulated on activation, normal T cell expressed and secreted, TNF-α, and TIMP-2 (Figures 1C,D; Table S2 in Supplementary Material). With regard to expression of the other 16 cytokines, the EGCG-treated sample showed downregulation, but this value was not significant (0.05 < *p* < 0.45) compared with that obtained for the LPS sample (Figures 1C,D). These results suggested that EGCG inhibited the release of inflammatory cytokines in the medium in THP-1-derived human macrophages during the immediate early stage of inflammation induced by LPS.

(−)-Epigallocatechin gallate had been shown to downregulate the mRNA levels of inflammatory cytokines in the intermediate or longer period of treatment of cells (>6 h). To ascertain whether the mRNA level of inflammatory cytokines could be attenuated in the immediate early period of EGCG treatment, the expression of major cytokines was detected on chips 1 h after EGCG addition using real-time PCR. Results showed that the mRNA levels of all measured inflammatory cytokines were decreased immediately after EGCG addition (Figure 1E). Taken together, these results suggested that EGCG could suppress the production of inflammatory cytokines induced by LPS in THP-1-derived human macrophages.

### EGCG Attenuated Inflammation Not Involved in the Classical Immune Pathway

Classical NF-κB and MAPK pathways have been proposed as the major mechanisms underlying inflammatory signaling. These pathways have also been proposed to mediate the effects of EGCG on inflammation in the intermediate (>6 h) or longer stages (≥12 h). To ascertain whether EGCG attenuated the immediate inflammatory response *via* the same mechanism, we designed and undertook intervention studies using EGCG.

**Figure 2:**
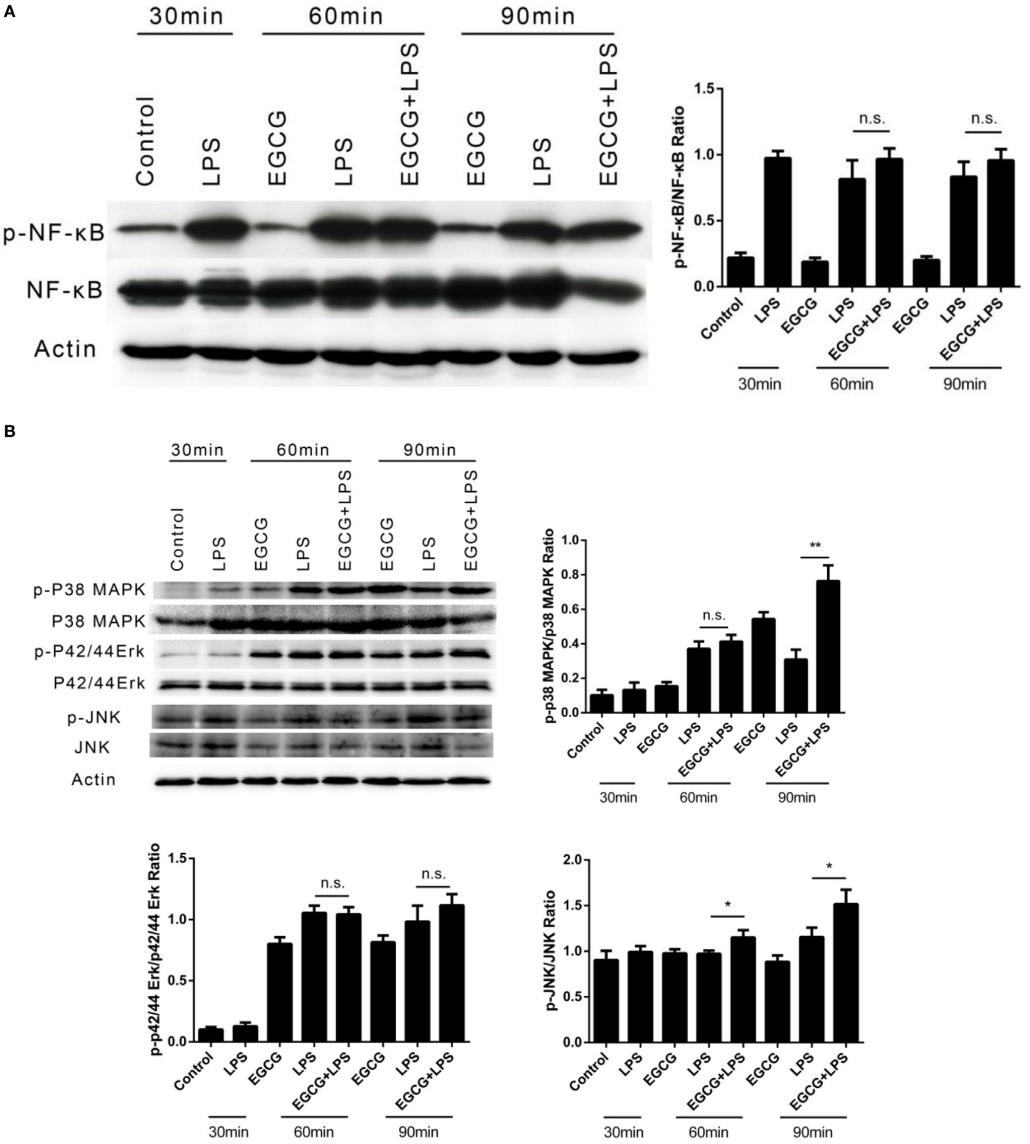
**(−)-Epigallocatechin gallate (EGCG) attenuated inflammation not involved in the classical immune pathway**. THP-1-derived macrophages were treated with lipopolysaccharide (LPS) (200 EU/mL) for 30, 60, and 90 min by pretreated EGCG (50 μg/mL, 30 min). The major immunological pathways nuclear factor-kappa B (NF-κB) **(A)** and MAPK [p38, p42/44, and c-Jun-*N*-terminal kinase (JNK)] **(B)** were detected. n.s., not significant.

The phosphorylation of NFκB and MAPK (i.e., p42/44, p38, and JNK) in THP-1-derived human macrophages was detected by western blotting after LPS treatment for 30, 60, and 90 min. EGCG was added 30 min before LPS. In contrast to the mechanisms described for EGCG upon inflammation, the phosphorylation of NFκB (Figure 2A), p42/44, and JNK (Figure 2B) was not inhibited by EGCG within the first 60 min, and the phosphorylation of p38 increased upon EGCG addition (Figure 2B). Sixty minutes after EGCG addition, NFκB phosphorylation remained unchanged. Moreover, the phosphorylation of ERK (p42/44) (Figure 2B) also increased upon EGCG addition. Increased phosphorylation has been shown to enhance the inflammatory response, and so the increased phosphorylation of MAPK induced by EGCG could not explain the attenuated inflammatory effects of EGCG and nor could NF-κB.

### EGCG Turns-Off Notch Signaling

**Figure 3:**
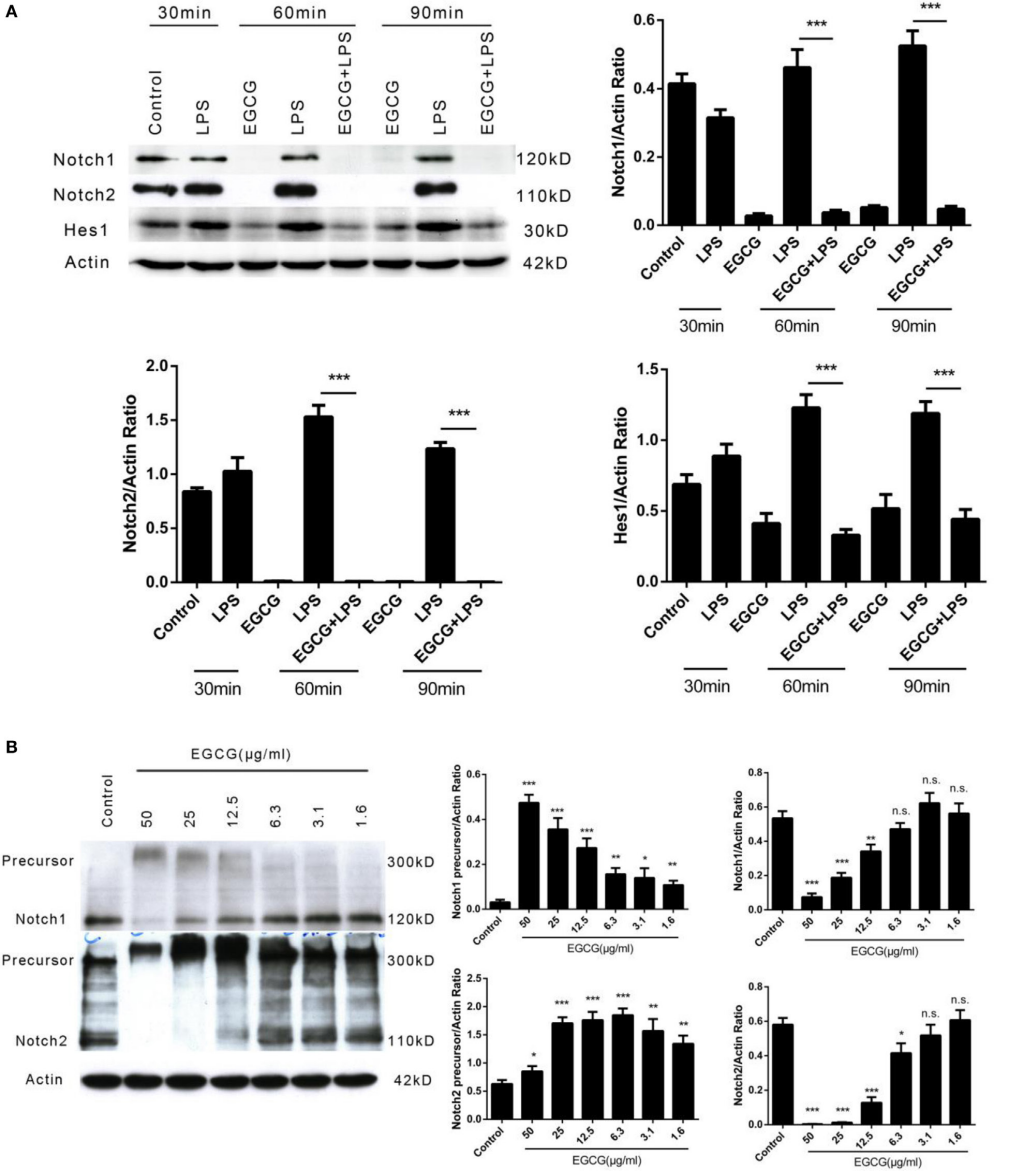

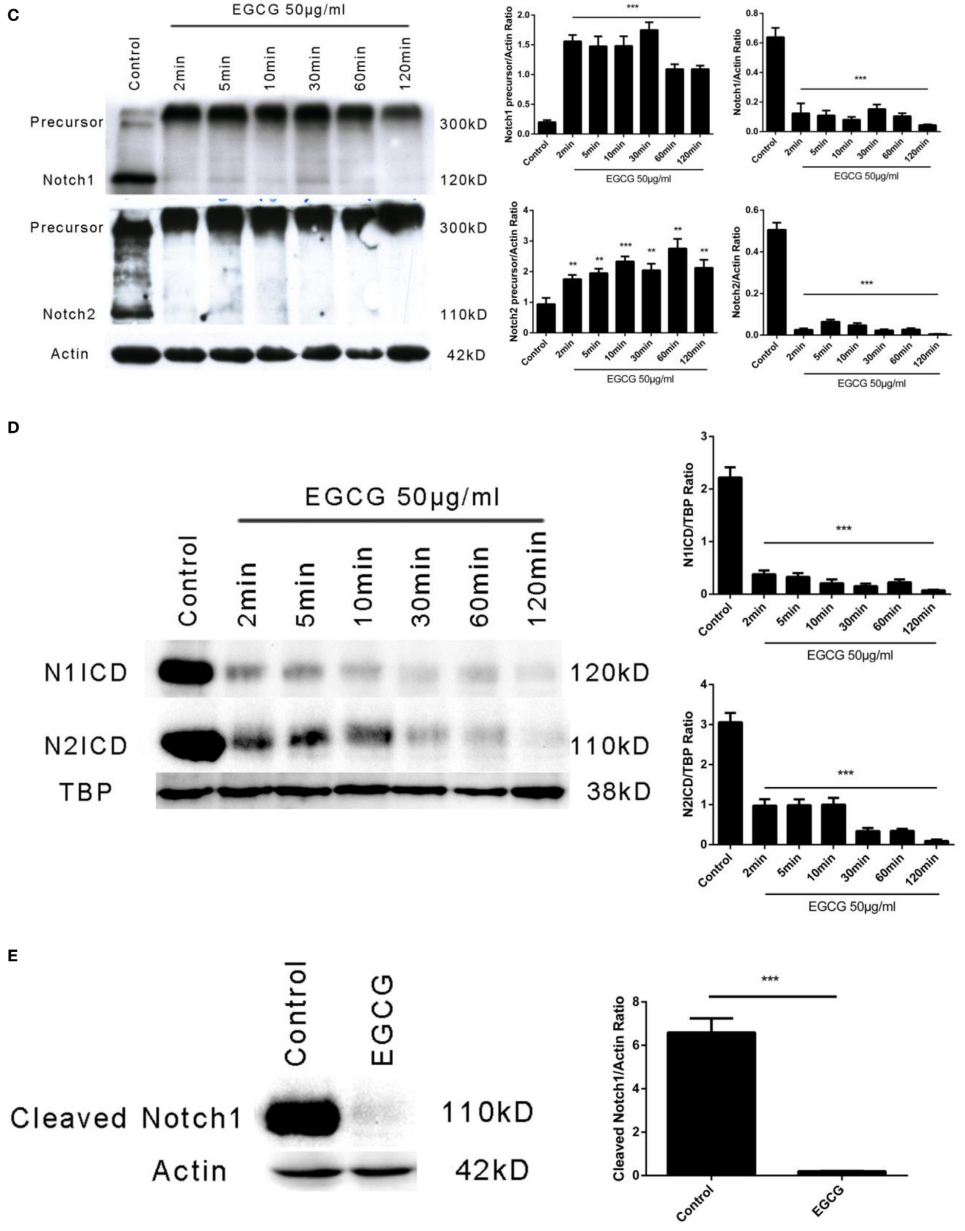

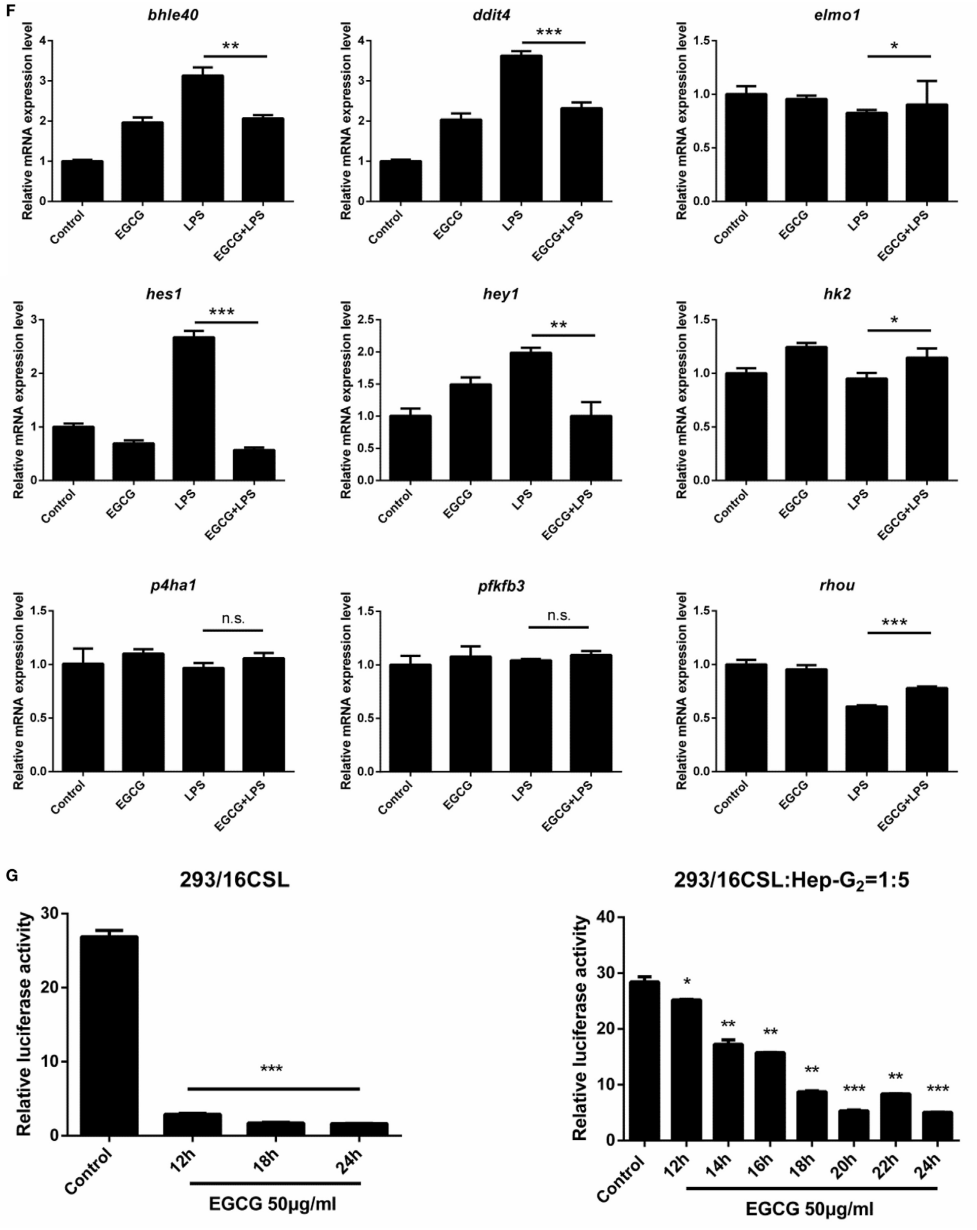

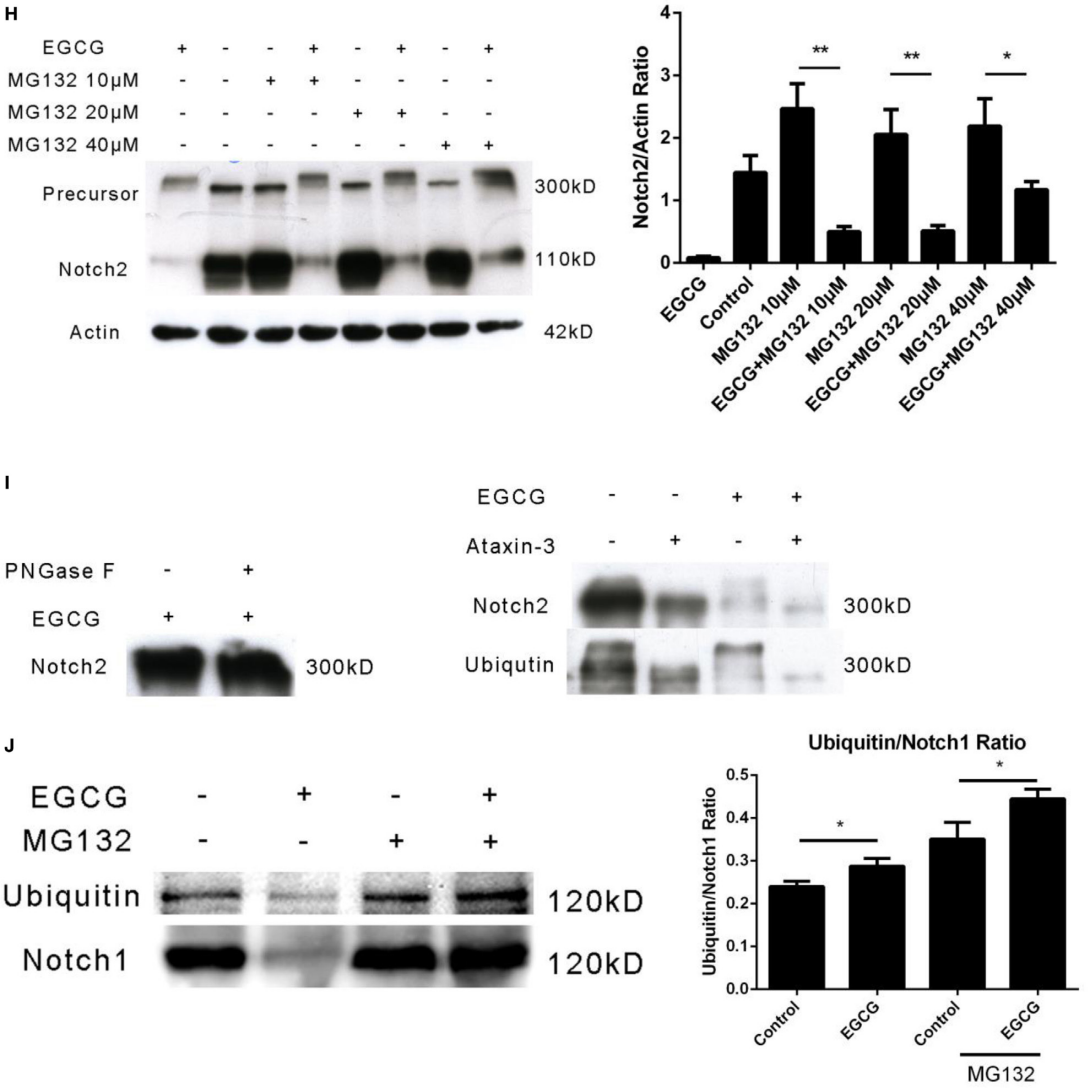
**(−)-Epigallocatechin gallate (EGCG) shuts off the Notch signal**. **(A)** THP-1-derived macrophages were treated with lipopolysaccharide (LPS) (200 EU/mL) for 30, 60, and 90 min by pretreated EGCG (50 μg/mL, 30 min). **(B)** THP-1-derived macrophages were treated with EGCG (50, 25, 12.5, 6.3, 3.1, and 1.6 μg/mL) for 30 min. **(C)** THP-1-derived macrophages were treated with EGCG (50 μg/mL) from 2 to 120 min. **(D)** THP-1-derived macrophages were treated with EGCG (50 μg/mL) from 2 to 120 min. Notch intercellular domain (NICD) of Notch1 and Notch2 located in the nucleus was detected, with TATA-binding protein (TBP) used as the internal control. **(E)** THP-1-derived macrophages were treated with EGCG (50 μg/mL) for 30 min, and lysates were analyzed by a western blotting probe with an antibody recognizing the epitope specific for active Notch1. **(F)** THP-1-derived macrophages were pretreated with EGCG (50 μg/mL) for 30 min and exposed to LPS (200 EU/mL) for 1 h. mRNA expression was measured by real-time polymerase chain reaction. **(G)** Notch report assays: 293/16CSL alone (left) and 293/16CSL with HepG2 coculture (right). **(H)** THP-1-derived macrophages were treated with EGCG (50 μg/mL) for 30 min with or without MG132 (10, 20, and 40 μM). **(I)** Immunoprecipitation was used to pull-down Notch from macrophage cell lysates. The Notch eluate was digested with PNGase F (left) and ATX-3 (right) and probed with the appropriate antibody. **(J)** Immunoprecipitation undertaken with antibody against the *C*-terminal of Notch1. The western blotting membrane was probed with an antibody against Notch1 or ubiquitin separately. Data are represented as mean ± SD (*n* = 3). Differences between the two groups were assessed by two-way ANOVA using GraphPad. Representatives of three independent experiments with similar results are shown. Results are represented as mean ± SD (*p < 0.05, **p < 0.01, and ***p < 0.001). n.s., not significant.

The Notch signaling pathway has a pivotal role in the development of multicellular organisms. Notch (specifically Notch1) is crucial for lymphocyte development (25). Emerging evidence has shown that the Notch pathway anticipates the immune response, including inflammation (26). Basal activation of Notch1 has been shown to be necessary for enhancement of the inflammatory response (27). To ascertain whether EGCG attenuates the inflammatory response *via* the Notch pathway immediately after its addition, we examined the Notch1 and Notch2 receptors, which are expressed on the surface of human macrophages at multiple time points shortly after EGCG treatment. We found that the number of mature Notch1 and Notch2 receptors was decreased from the cell surface at all time points after EGCG treatment and that this process was not dependent on LPS stimulation (Figure 3A). Further studies showed that mature Notch removed the effects of EGCG in a concentration-dependent manner (Figure 3B) and that this decreased response was rapid (2 min) (Figure 3C). Simultaneously, EGCG treatment accelerated the degradation of the nuclear Notch intercellular domain (NICD), the active form of Notch, in 2 min (Figure 3D). In accordance with this result, a western blotting probe with a specific antibody against the new epitope formed after Notch1 cleavage showed that degradation of the active form of Notch1 was promoted by EGCG (Figure 3E). These results suggested that the basal signaling of Notch was turned off immediately by EGCG treatment. In accordance with these results, expression of Hes1 (a typical Notch-regulated gene) was downregulated by EGCG (Figure 3A). To confirm this result, expression of Notch target genes was evaluated using real-time PCR. We found that expression of some typical Notch target genes was downregulated by EGCG (Figure 3F), but not all of them. This finding may have been due to the complexity of gene transcription, which can be influenced by many factors. To evaluate the effects of EGCG treatment on gene expression of Notch alone, a dual luciferase reporter assay was employed. This assay involves use of the luciferase gene, which is controlled by 16×CSL, which contains 16 major Notch-regulated elements (CSL) connected in a series. Results showed that only treatment with EGCG could decrease Notch-related luciferase signaling in 293T cells and HepG2/293T cocultures (Figure 3G).

To understand the mechanism of Notch suppression induced by EGCG, the proteasome inhibitor MG132 was used to pretreat macrophages before EGCG addition. Notch degradation was inhibited partially by increasing (10–40 μM) concentrations of MG132 (Figure 3H). This result showed that MG132 inhibited the Notch degradation induced by EGCG. Western blotting demonstrated that pre-Notch1/2 accumulated immediately after EGCG treatment and that the molecular weight of pre-Notch1/2 increased over time (Figures 3B,C). The major change in molecular weight occurred with posttranslational modification of the protein, including glycosylation and polyubiquitination. Thus, the immunoprecipitated Notch2 from THP-1-derived macrophages was treated separately with PNGase F (which removes *N*-linked polysaccharides from the glycosylated protein) or ataxin-3 (an enzyme that removes ubiquitin from the target protein). The increased molecular weight of the accumulated precursor of Notch2 after EGCG treatment (Figures 3B,C) was decreased upon treatment with ataxin-3, but not PNGase F (Figure 3I). This result confirmed that pre-Notch was highly ubiquitinated (but not glycosylated) in EGCG-treated cells. In accordance with the MG132-based inhibition of Notch degradation induced by EGCG, the Notch mature/active form was overubiquitinated after EGCG treatment as demonstrated by an immunoprecipitation–western blotting experiment using an antibody against Notch or ubiquitin separately (Figure 3J). These results demonstrated that EGCG induced switching off of the Notch signal by inducing Notch degradation *via* a proteasome pathway.

### Notch Is a New Target of EGCG Independent of 67LR

**Figure 4:**
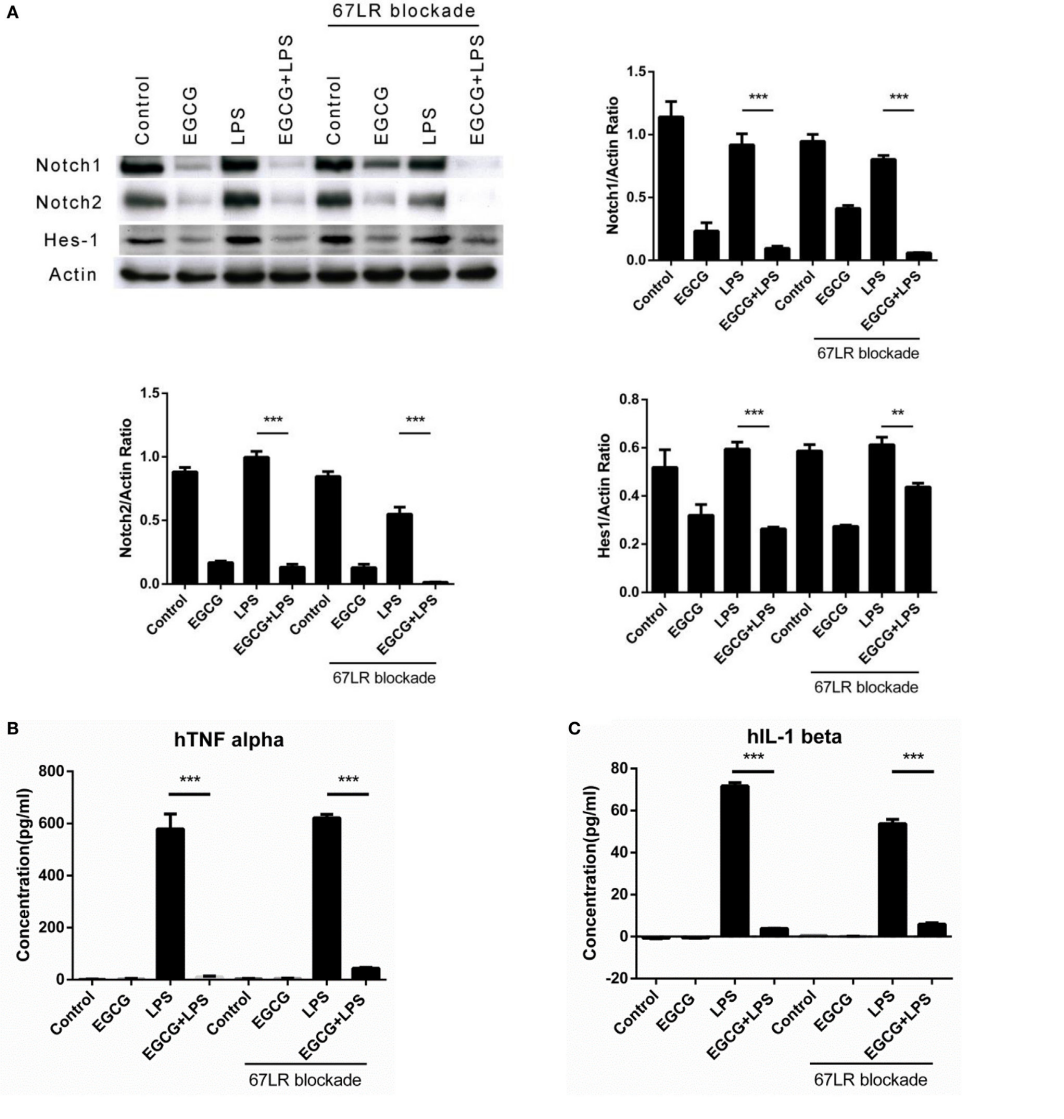

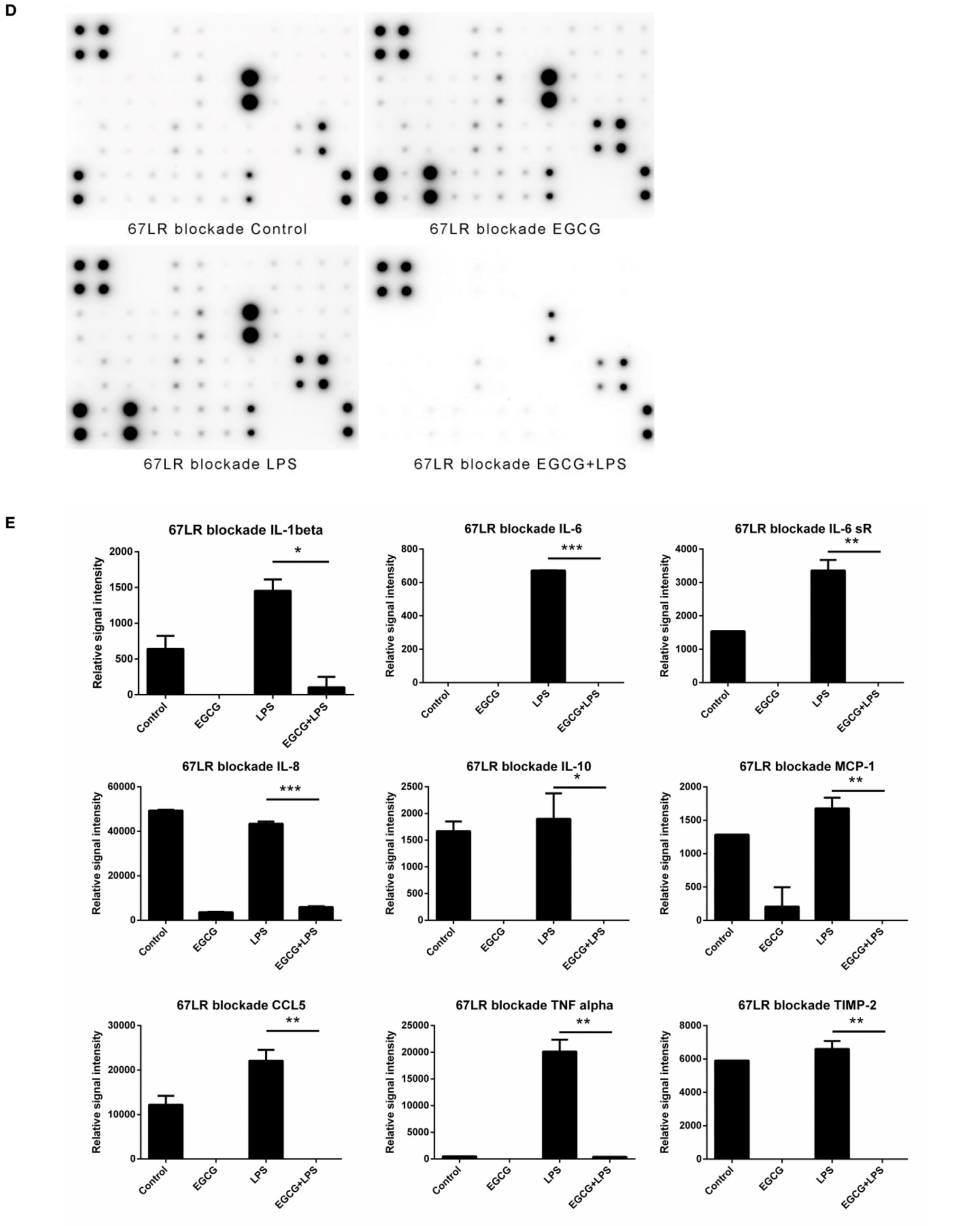

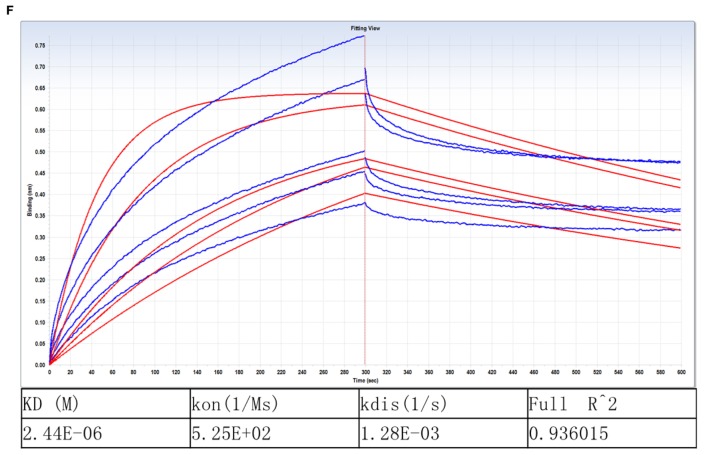
**Notch is a new target of (−)-epigallocatechin gallate (EGCG) independent of the 67-kDa laminin receptor (67LR)**. THP-1-derived macrophages were preincubated with 67LR antibody (5 μg/mL) for 1 h and then treated with EGCG (50 μg/mL) for 30 min. Cell lysates were probed with Notch1/2 and hairy and enhancer of split-1 (Hes1). **(A)** THP-1-derived macrophages were pretreated with EGCG (50 μg/mL) for 30 min before exposure to lipopolysaccharide (LPS) (200 EU/mL) for 3 h. Cell lysates were probed with Notch1/2 and Hes1 **(A)**. Expression of the inflammatory cytokines in THP-1-derived macrophages in the culture medium was measured by enzyme-linked immunosorbent assay **(B,C)** and RayBio C-Series human inflammation antibody array **(D)**. Chips were scanned and analyzed **(E)**. **(F)** Interaction between EGCG and NRR1 was measured on an Octet Red96 system with association and dissociation for 300 s, respectively. The concentration of EGCG used was a double dilution starting from 40 μM. Kinetic parameters and affinities were calculated from a non-linear global fit. Data are represented as mean ± SD (*n* = 3). Differences between the two groups were assessed by two-way ANOVA using GraphPad. Representatives of three independent experiments with similar results are shown. Results are represented as mean ± SD (*p < 0.05, **p < 0.01, and ***p < 0.001).

67-kDa laminin receptor has been proposed to be a cell surface target for EGCG to mediate its biologic activity (11). Scholars have reported that EGCG downregulates the inflammatory response in mouse macrophages *via* 67LR (28). However, those studies confirmed the effects at long time points (typically >6 h). To ascertain whether 67LR mediated the effects of EGCG on Notch and inflammation in the immediate early stage in THP-1-derived human macrophages, cells were pretreated for 1 h with the anti-67LR antibody MLuC5 (5 μg/mL), which is known to block the interaction between EGCG and 67LR. MluC5 blockade did not affect the suppression of Notch by EGCG (Figure 4A) or expression of the typical Notch-regulated protein Hes1 (Figure 4A). The effects of EGCG on the NF-κB (Figure S3A in Supplementary Material) and MAPK (Figure S3B in Supplementary Material) pathways were unaltered by MluC5 blockade 30 min after EGCG addition. To study the inflammatory effects of EGCG at the immediate early stage, MLuC5-treated macrophages were pretreated for 30 min with EGCG before LPS exposure. Production of TNF-α and IL-1β in culture supernatants from human macrophages induced by LPS was inhibited markedly by EGCG treatment in anti-67LR antibody-treated cells and control cells (Figures 4B,C). To determine the influence of 67LR blockade on the inflammation-attenuated effects of EGCG in human macrophages, we examined the inflammatory cytokines released in culture supernatants 3 h after LPS treatment using a human inflammation antibody array (RayBio). Expression of 27 of 40 inflammatory factors induced by LPS stimulation, as well as TNF-α and IL-1β (Figures 4B,C; Table S3 in Supplementary Material), was reduced significantly (*p* < 0.05, *p* < 0.01, or *p* < 0.001) by EGCG when macrophages were blocked by MluC5 (Figures 4D,E). These results were comparable to those of the non-blocking experiments (Figures 1C,D; Table S3 in Supplementary Material). The expression of major inflammatory cytokines downregulated by EGCG, such as TNF-α, IL-1β, IL-6, IL-8, IL-10, MCP-1, IL-6sR, CCL5/RANTES, and TIMP-2, was not affected by MluC5 pretreatment.

To measure a possible direct interaction between Notch and EGCG, we prepared the human NRR1. The NRR1 is located outside the cell membrane and has been considered to have a key role in Notch1 activation. Binding assays showed that NRR1 had a direct interaction with EGCG and that the affinity of EGCG to NRR1 was 2.44 × 10^–6^ (Figure 4F).

Taken together, these results demonstrated that 67LR had no influence on the inflammation-attenuated effects of EGCG at the immediate early stage. Thus, Notch is a new target of EGCG.

### Knockdown of Notch 1/2 Impaired the Effects of EGCG on Inflammation

**Figure 5:**
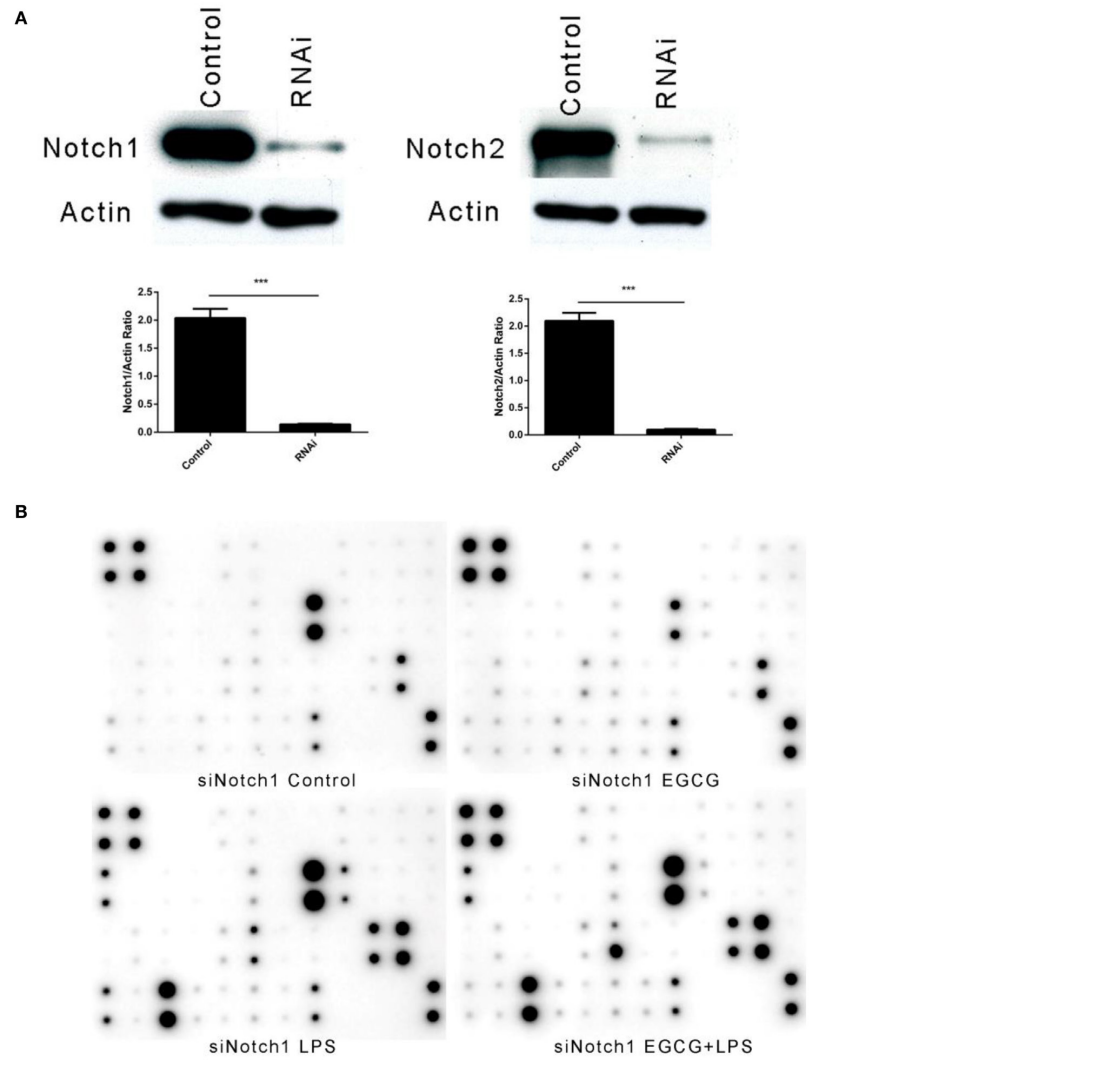

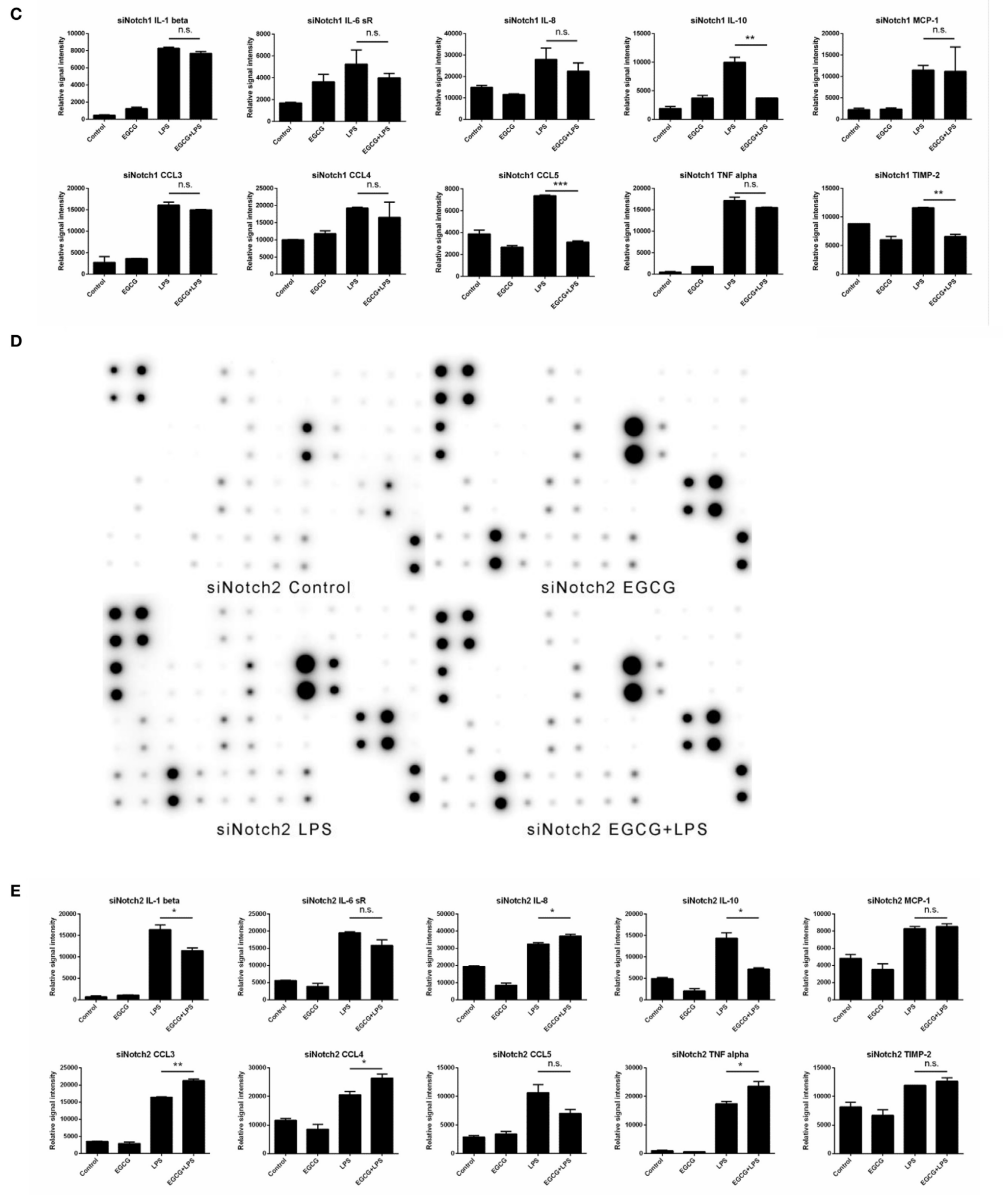

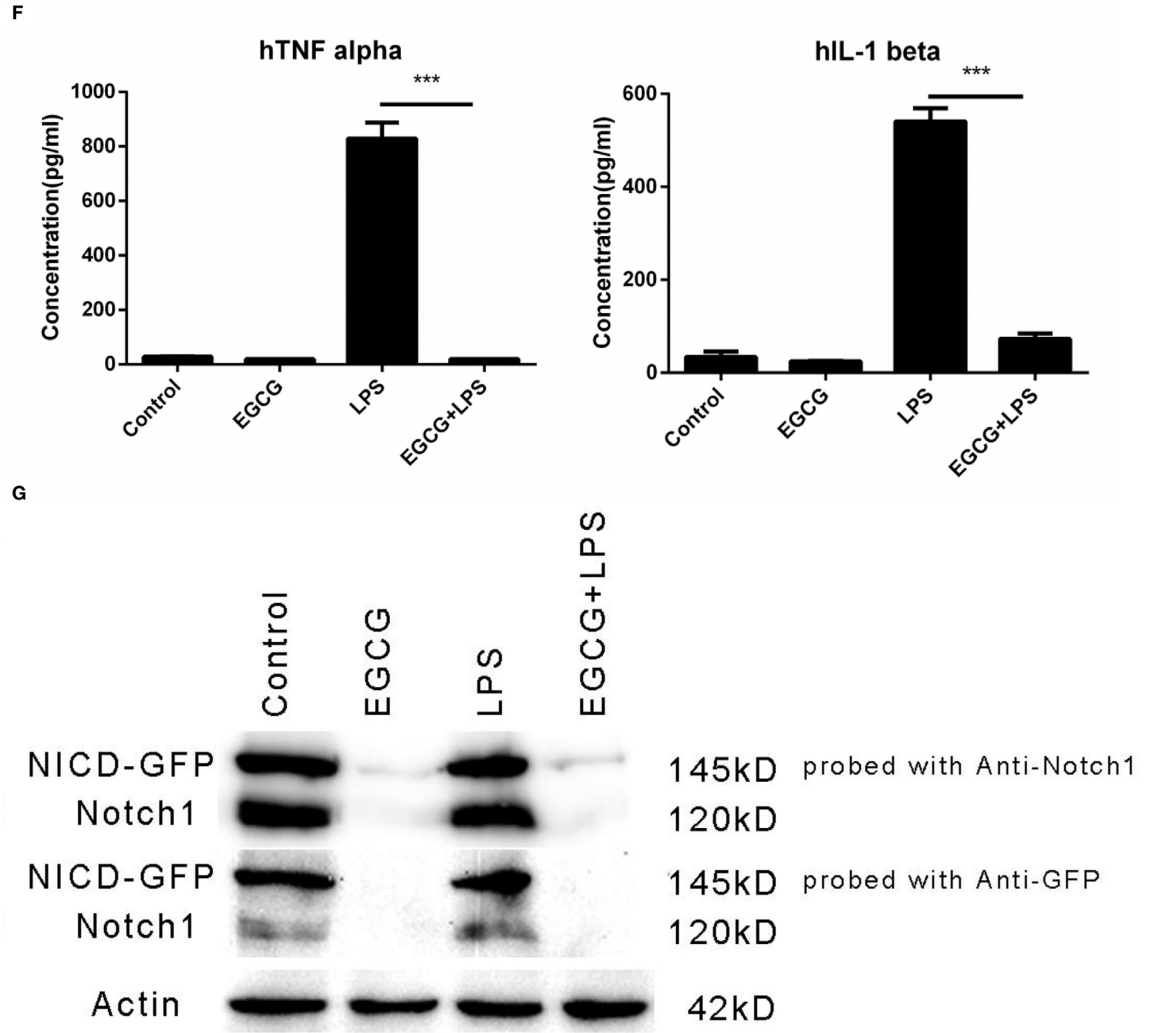
**Notch1/2 knockdown impaired the effects of (−)-epigallocatechin gallate (EGCG) on inflammation**. After knockdown of Notch1 or Notch2 through infection by lentivirus particles, inflammation experiments were done the same way as for wild-type THP-1-derived macrophages. Knockdown of Notch1 or Notch2 was confirmed by western blotting **(A)**. Notch1/2 knockdown macrophages were pretreated with EGCG (50 μg/mL) for 30 min before exposure to lipopolysaccharide (LPS) (200 EU/mL) for 3 h. Expression of the inflammatory cytokines in the culture medium was measured by a RayBio C-Series human inflammation antibody array: Notch1 knockdown **(B)** and Notch2 knockdown **(D)**. Chips were scanned and analyzed: Notch1 knockdown **(C)** and Notch2 knockdown **(E)**. Notch intercellular domain (NICD)-green fluorescent protein (GFP)-overexpressed macrophages were pretreated with EGCG (50 μg/mL) for 30 min and exposed to LPS (200 EU/mL) for 3 h. Levels of tumor necrosis factor (TNF)-α and interleukin (IL)-1β in the culture medium were measured by enzyme-linked immunosorbent assay **(F)**, and cell lysates were analyzed by western blotting and a probe with antibody against Notch1 or GFP separately **(G)**. Data are represented as mean ± SD (*n* = 3). Differences between the two groups were assessed by two-way ANOVA using GraphPad. Representatives of three independent experiments with similar results are shown. Results are represented as mean ± SD (*p < 0.05, **p < 0.01, and ***p < 0.001). n.s., not significant.

To investigate whether the effects of EGCG on cellular responses to LPS stimulation are dependent on Notch, we undertook shRNA knockdown of Notch 1/2 in THP-1 cells. THP-1 cells were infected with Notch1/2 shRNA lentiviral particles (Santa Cruz Biotechnology) and screened with puromycin (5 μg/mL) to silence expression of Notch1 or Notch2. Notch 1/2 silencing was confirmed by western blotting (Figure 5A). Notch 1/2-silenced THP-1 cells were treated with PMA (10 ng/mL) for 48 h to obtain macrophages that were similar to wild-type macrophages. Notch 1/2-silenced macrophages were stimulated with LPS (200 EU/mL) and pretreated (or not) for 30 min with EGCG. Expression of the inflammatory cytokines in samples 3 h after LPS addition was measured using a human inflammation antibody array (RayBio). Results showed that Notch1/2 knockdown impaired the attenuated effects of EGCG on release of inflammatory cytokines (Figures 5B–E). Further analyses of the inflammation array results revealed that Notch1 knockdown blocked the attenuated effects of EGCG in 30 of 40 of the inflammatory cytokines and that knockdown of Notch2 blocked the attenuated effects of EGCG in 31 of the 40 inflammatory cytokines (Figures 5B–E; Tables S3 and S4 in Supplementary Material). These results demonstrated that Notch1/2 knockdown greatly impaired the inflammation-attenuated effects of EGCG and that Notch1/2 is a major target for EGCG for reduction of the inflammatory response in the early stage.

We wished to evaluate whether overexpression of the active form of Notch could impair the inflammation-attenuated effects of EGCG. Hence, NICD-green fluorescent protein, the constitutively active form of Notch1, was overexpressed in THP-1 cells by transfection, and the cells were sorted by fluorescence-activated cell sorting. However, transfection of the active form of Notch did not erase the effects of EGCG (Figure 5F) because EGCG treatment led to degradation of the original form and active form of Notch (Figure 5G). These data suggested that rapid degradation of Notch (including the basal active NICD) was induced by EGCG treatment. Such treatment also accelerated degradation of the active form of Notch (NICD-GFP).

### EGCG Suppressed LPS-Induced Inflammation in Human Primary Macrophages

**Figure 6:**
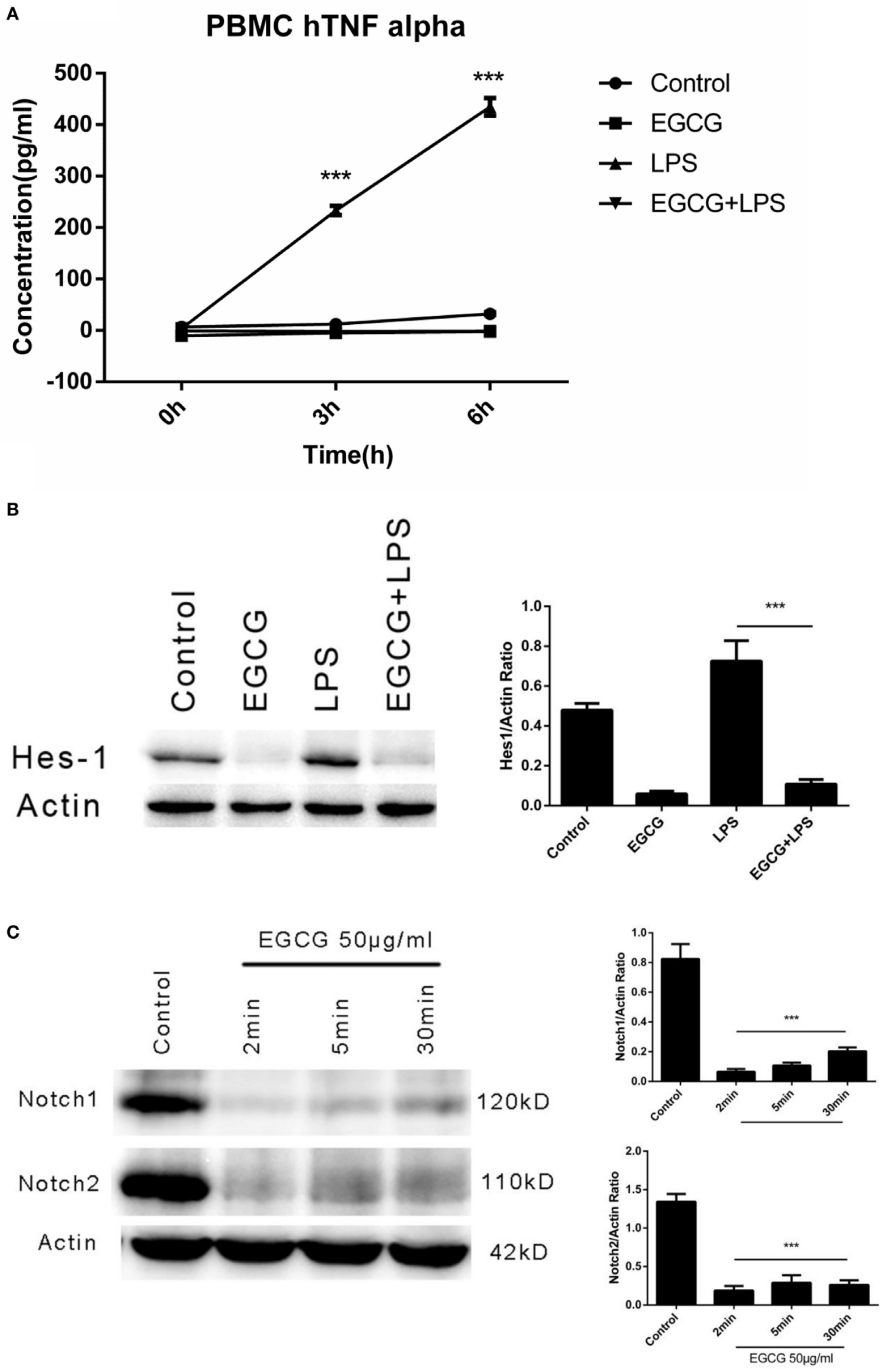
**(−)-Epigallocatechin gallate (EGCG) suppressed lipopolysaccharide (LPS)-induced inflammation in human primary macrophages**. Human primary macrophages derived from peripheral blood mononuclear cells (PBMCs) cultured with autologous serum were pretreated with EGCG (50 μg/mL) for 30 min and exposed to LPS (200 EU/mL) for 3 h. Expression of inflammatory cytokines was measured by enzyme-linked immunosorbent assay **(A)**. Cell lysates 30 min after EGCG treatment were analyzed by western blotting and probed with hairy and enhancer of split-1 (Hes1) antibody **(B)**. Cell lysates from time course experiments of EGCG treatment were analyzed by western blotting and probed with a Notch1/2 antibody **(C)**.

We demonstrated that EGCG attenuated the LPS-induced inflammation response *via* Notch1/2 in THP-1-derived macrophages. However, the THP-1 cell is derived from a patient with acute monocytic leukemia, and so it may demonstrate different mechanisms with regard to regulation of the inflammatory response. To evaluate the effects of EGCG on normal human cells, we examined the anti-inflammatory effect of EGCG on primary human macrophages differentiated from PBMCs collected from healthy human donors. Human primary macrophages cultured with 5% autologous serum were used in LPS/EGCG experiments. Similar to THP-1-derived macrophages, primary human macrophages were pretreated for 30 min with EGCG before LPS exposure. In accordance with the findings obtained in THP-1 cells, TNF-α production in the culture supernatants obtained from primary human macrophages was increased significantly (*p* < 0.001) with LPS treatment, and LPS-induced production of inflammatory cytokines was inhibited significantly (*p* < 0.001) in EGCG-pretreated cells (Figure 6A). EGCG treatment also suppressed the expression of Notch1/2 and the typical Notch target protein Hes1 (Figure 6B). The time course in EGCG-treated human primary macrophages showed that EGCG suppressed Notch1/2 at 2 min (Figure 6C). Taken together, these results showed that human primary macrophages obtained from PBMCs exhibited the same effects as THP-1-derived macrophages upon EGCG treatment.

## Discussion

The inflammatory response, specifically that induced by LPS, can be elicited in minutes and even seconds (29, 30). The phosphorylation and nuclear translocation of the major mediators of inflammation, such as NF-κB, can be detected within 1 min (31, 32). Upregulated expression of the genes of inflammatory cytokines can be observed within several minutes using real-time PCR. Moreover, the release of the “first round” of inflammatory cytokines, such as TNF-α, can be measured by ELISA 2–3 h after LPS addition. Three hours later, the released cytokines in the medium can engage with receptors on the cell membranes of cells to initiate the “second round” of the cellular response, including inflammation. Taken together, these events complicate examination of the mechanism underlying regulation of the inflammatory response and interference by special reagents. Thus, it is important to uncover the original mechanism or target in the immediate early stage.

(−)-Epigallocatechin gallate is a major bioactive compound found in Chinese green tea. It has been shown to attenuate the inflammatory response (33). The mechanisms of EGCG upon inflammation have been explored in detail. However, those studies focused on the intermediate or longer stages, which is typically >6 h or overnight, following EGCG treatment (34, 35). EGCG has been shown to downregulate the phosphorylation of key regulators of the classical pathway of the inflammatory response, such as NF-κB. In addition, 67LR (which has been proposed to be the receptor for EGCG identified from mouse cell membranes) has been shown to mediate downregulation of the anti-inflammation effects of EGCG. However, those studies also focused on the physiologic responses of cells ≥6 h after EGCG treatment. Those data suggest that EGCG treatment interferes with the mediator proteins in the signal transduction pathway, but the original mechanism of EGCG treatment is not known.

Here, we studied the immediate early response of macrophages after EGCG addition and revealed the mechanism of action of Notch. First, the inflammation-attenuated effects of EGCG in the immediate early stage were confirmed by measuring cytokine levels in the culture medium and cytokine mRNA levels in macrophages. Induction of inflammatory cytokines can be measured only 3 h after LPS addition, so ELISA and human inflammation chips were employed. EGCG reduced the expression of 24 of 40 inflammatory cytokines, and mRNA levels decreased in 9 of 10 genes 1 h after LPS addition. These results confirm that EGCG attenuates the LPS-induced inflammatory response in the immediate early stage. With regard to classical inflammation pathways, NF-κB and MAPK were examined to ascertain whether they are the major mediators of the EGCG-regulated inflammatory response in the immediate early stage. Unexpectedly, the NF-κB and MAPK pathways were not involved in reducing the anti-inflammatory effects of EGCG. However, increased phosphorylation of p38 and ERK was observed after EGCG addition (Figure 2B). This finding conflicts with data showing that MAPK is inhibited by EGCG (36, 37). The active form of the MAPK pathway is considered to increase the inflammatory response, even though it is a weaker regulator compared with NF-κB in macrophages. The increased activation of MAPK by EGCG in the early stage may be due to an unknown mechanism of EGCG. These data suggest the EGCG may target two signaling pathways that regulate inflammation at the early stage: activation of MAPK and shutting down Notch. In accordance with this hypothesis, EGCG treatment increased the basal inflammatory response due to MAPK activation when Notch1 was knocked down.

Based on the potential relationship between EGCG/Notch shown previously and knowledge of the role of Notch in the inflammatory process, Notch signaling was examined for mature Notch receptors and the active form of Notch, NICD (which is located in the nucleus). We found that the Notch receptor on cell membranes and the basal signal of NICD in the nucleus were erased 2 min after EGCG addition. Further study showed that the Notch signal shut-off by EGCG was concentration dependent and was not dependent on LPS treatment. Consistent with the suppression of Notch signaling, the mRNA levels of typical Notch-regulated genes were downregulated following EGCG treatment, including the protein levels of Notch target genes such as HES-1. These results demonstrate that basal Notch signaling was suppressed by EGCG. To evaluate the Notch-suppressive effect of EGCG in live cells, we undertook a Notch reporter assay in 293T cells transfected with pCMV/R-Luc and a firefly luciferase Notch reporter plasmid. We constructed them by placing the multiple NICD binding sequence (16CSL) upstream of the mini-promoter to control expression of the firefly luciferase gene. Coculture of 293T cells with HepG2 (used to provide the ligand for Notch activation) and 293T cells alone showed that Notch signaling was suppressed upon EGCG treatment. The proteasome inhibitor MG132 inhibited the Notch degradation induced by EGCG. Consistent with this result, the increase in molecular weight of pre-Notch2 induced by EGCG was confirmed due to multiple ubiquitination. Also, the ubiquitination level of mature/active Notch was increased by EGCG treatment. These results show that EGCG suppresses Notch signaling *via* a proteasome pathway.

We revealed that Notch localized on cell membranes can be a target of EGCG to attenuate the immune response in the immediate early stage. However, another surface protein, 67LR, has been shown to mediate the attenuated effect of the immune response by EGCG. To determine whether the effects of EGCG on Notch are 67LR dependent, the 67LR-blocking antibody MluC5 (which has been shown to block the 67LR-dependent effects of EGCG) was used in EGCG/Notch experiments to block the potential effects of EGCG/67LR on Notch. 67LR blockade did not impair the effects of EGCG on the immune response and Notch. These results also show that the effects of Notch by EGCG are not dependent on 67LR and that EGCG interacts with the NRR1 located outside the cell membrane of mature Notch1 at high affinity. Hence, Notch is a new target of EGCG that can modulate the immune response at the immediate early stage.

To evaluate the necessity of Notch in the immune response induced by EGCG, Notch1/2 was knocked down by infecting THP-1 cells with Notch1/2 shRNA lentiviral particles. The EGCG/Notch experiments done in macrophages derived from Notch1/2 knockdown THP-1 cells showed that knockdown of Notch1/2 lowered the strength of the immune response induced by LPS and impaired the attenuated effects of EGCG in the immediate early stage. The NICD overexpression experiment didn’t show new results due to the transfected NICD is promoted to degradation by the EGCG treatment also. A basal active Notch signal is necessary for amplification of the inflammatory response, so Notch1/2 knockdown can decrease the severity of inflammation. Also, EGCG cannot lower the inflammation further if Notch1/2 has been knocked down (Figure S4 in Supplementary Material). Hence, we can conclude that Notch is the primary target of EGCG to downregulate inflammation in wild-type macrophages. Taken together, these results are consistent with previous reports on the function of Notch in the immune response and provide new information. We also carried out the EGCG/Notch experiments on primary human macrophages differentiated from PBMCs obtained from healthy donors. Those results confirmed the effects of EGCG on Notch and the immune response.

Interestingly, we showed that the Notch shut-off by EGCG is not dependent on LPS treatment and last from minutes to over 6–18 h during the EGCG treatment (Figure S5 in Supplementary Material). These results suggest that the interaction between Notch and EGCG is likely to be a conservative biochemical reaction in other types of cells, not just macrophages. Attenuation of the inflammatory response could be just one of the effects of EGCG/Notch in macrophages. There may be diverse effects upon different cell types by EGCG due to the multiple functions of the Notch receptor (38).

In summary, we examined the immediate early mechanism of EGCG on the inflammatory response induced by LPS in human macrophages. We found that shutting off of Notch signaling is a key event in the inflammation–attenuation effects induced by EGCG in the immediate early stage.

## Author Contributions

JS, XW, and SH conceived and designed the experiments. TW, ZX, XL, YW, CF, SS, CL, HY, HW, and LY performed the experiments and analyzed the data. JS, XW, and SH contributed reagents/materials/analysis tools. TW and XL wrote the manuscript. All authors read and approved the final manuscript.

## Conflict of Interest Statement

The authors declare that the research was conducted in the absence of any commercial or financial relationships that could be construed as a potential conflict of interest.

## References

[1] Coussens LM Werb Z. Inflammation and cancer. Nature (2002) 420(6917):860–7. 10.1038/nature01322 12490959 PMC2803035

[2] Wellen KE Hotamisligil GS Inflammation, stress, and diabetes. J Clin Invest (2005) 115(5):1111–9. 10.1172/JCI25102 15864338 PMC1087185

[3] Kamada N Seo SU Chen GY Nunez G. Role of the gut microbiota in immunity and inflammatory disease. Nat Rev Immunol (2013) 13(5):321–35. 10.1038/nri3430 23618829

[4] Heneka MT Carson MJ El Khoury J Landreth GE Brosseron F Feinstein DL Neuroinflammation in Alzheimer’s disease. Lancet Neurol (2015) 14(4):388–405. 10.1016/S1474-4422(15)70016-5 25792098 PMC5909703

[5] Tabas I Glass CK Anti-inflammatory therapy in chronic disease: challenges and opportunities. Science (2013) 339(6116):166–72. 10.1126/science.1230720 23307734 PMC3608517

[6] Newman DJ Cragg GM. Natural products as sources of new drugs over the 30 years from 1981 to 2010. J Nat Prod (2012) 75(3):311–35. 10.1021/np200906s 22316239 PMC3721181

[7] Villa FA Gerwick L. Marine natural product drug discovery: leads for treatment of inflammation, cancer, infections, and neurological disorders. Immunopharmacol Immunotoxicol (2010) 32(2):228–37. 10.3109/08923970903296136 20441539

[8] Peairs A Dai R Gan L Shimp S Rylander MN Li L Epigallocatechin-3-gallate (EGCG) attenuates inflammation in MRL/lpr mouse mesangial cells. Cell Mol Immunol (2010) 7(2):123–32. 10.1038/cmi.2010.1 20140007 PMC4001894

[9] Zhong Y Chiou YS Pan MH Shahidi F. Anti-inflammatory activity of lipophilic epigallocatechin gallate (EGCG) derivatives in LPS-stimulated murine macrophages. Food Chem (2012) 134(2):742–8. 10.1016/j.foodchem.2012.02.172 23107686

[10] Syed DN Afaq F Kweon MH Hadi N Bhatia N Spiegelman VS Green tea polyphenol EGCG suppresses cigarette smoke condensate-induced NF-kappaB activation in normal human bronchial epithelial cells. Oncogene (2007) 26(5):673–82. 10.1038/sj.onc.1209829 16862172

[11] Tachibana H Koga K Fujimura Y Yamada K A receptor for green tea polyphenol EGCG. Nat Struct Mol Biol (2004) 11(4):380–1. 10.1038/nsmb743 15024383

[12] Luo JL Maeda S Hsu LC Yagita H Karin M. Inhibition of NF-kappaB in cancer cells converts inflammation-induced tumor growth mediated by TNFalpha to TRAIL-mediated tumor regression. Cancer Cell (2004) 6(3):297–305. 10.1016/j.ccr.2004.08.012 15380520

[13] Johnson GL Lapadat R Mitogen-activated protein kinase pathways mediated by ERK, JNK, and p38 protein kinases. Science (2002) 298(5600):1911–2. 10.1126/science.1072682 12471242

[14] Kopan R Ilagan MX. The canonical Notch signaling pathway: unfolding the activation mechanism. Cell (2009) 137(2):216–33. 10.1016/j.cell.2009.03.045 19379690 PMC2827930

[15] Minter LM Turley DM Das P Shin HM Joshi I Lawlor RG Inhibitors of gamma-secretase block in vivo and in vitro T helper type 1 polarization by preventing Notch upregulation of Tbx21. Nat Immunol (2005) 6(7):680–8. 10.1038/ni1209 15991363

[16] Yuan JS Kousis PC Suliman S Visan I Guidos CJ. Functions of Notch signaling in the immune system: consensus and controversies. Annu Rev Immunol (2010) 28:343–65. 10.1146/annurev.immunol.021908.132719 20192807

[17] De Obaldia ME Bell JJ Wang X Harly C Yashiro-Ohtani Y DeLong JH T cell development requires constraint of the myeloid regulator C/EBP-alpha by the Notch target and transcriptional repressor Hes1. Nat Immunol (2013) 14(12):1277–84. 10.1038/ni.2760 24185616 PMC4038953

[18] Smale ST. Selective transcription in response to an inflammatory stimulus. Cell (2010) 140(6):833–44. 10.1016/j.cell.2010.01.037 20303874 PMC2847629

[19] Takeuchi O Akira S. Toll-like receptors; their physiological role and signal transduction system. Int Immunopharmacol (2001) 1(4):625–35. 10.1016/S1567-5769(01)00010-8 11357875

[20] Yin J Huang F Yi Y Yin L Peng D. EGCG attenuates atherosclerosis through the Jagged-1/Notch pathway. Int J Mol Med (2016) 37(2):398–406. 10.3892/ijmm.2015.2422 26648562

[21] Xie H Sun J Chen Y Zong M Li S Wang Y. 2b.06: epigallocatechin-3-gallate attenuates uric acid-induced inflammatory responses and oxidative stress by modulating Notch pathway. J Hypertens (2015) 33(Suppl 1):e23. 10.1097/01.hjh.0000467411.86784.c1 26539255 PMC4619967

[22] Gu LT Yang J Su SZ Liu WW Shi ZG Wang QR. Green tea polyphenols protects cochlear hair cells from ototoxicity by inhibiting Notch signalling. Neurochem Res (2015) 40(6):1211–9. 10.1007/s11064-015-1584-3 25896296

[23] Jin H Gong W Zhang C Wang S. Epigallocatechin gallate inhibits the proliferation of colorectal cancer cells by regulating Notch signaling. Onco Targets Ther (2013) 6:145–53. 10.2147/OTT.S40914 23525843 PMC3596123

[24] Park EK Jung HS Yang HI Yoo MC Kim C Kim KS. Optimized THP-1 differentiation is required for the detection of responses to weak stimuli. Inflamm Res (2007) 56(1):45–50. 10.1007/s00011-007-6115-5 17334670

[25] Allman D Punt JA Izon DJ Aster JC Pear WS. An invitation to T and more: Notch signaling in lymphopoiesis. Cell (2002) 109(Suppl):S1–11. 10.1016/S0092-8674(02)00689-X 11983148

[26] Radtke F Fasnacht N Macdonald HR. Notch signaling in the immune system. Immunity (2010) 32(1):14–27. 10.1016/j.immuni.2010.01.004 20152168

[27] Hu X Chung AY Wu I Foldi J Chen J Ji JD Integrated regulation of toll-like receptor responses by Notch and interferon-gamma pathways. Immunity (2008) 29(5):691–703. 10.1016/j.immuni.2008.08.016 18976936 PMC2585039

[28] Hong Byun E Fujimura Y Yamada K Tachibana H. TLR4 signaling inhibitory pathway induced by green tea polyphenol epigallocatechin-3-gallate through 67-kDa laminin receptor. J Immunol (2010) 185(1):33–45. 10.4049/jimmunol.0903742 20511545

[29] Arai KI Lee F Miyajima A Miyatake S Arai N Yokota T Cytokines: coordinators of immune and inflammatory responses. Annu Rev Biochem (1990) 59:783–836. 10.1146/annurev.bi.59.070190.004031 1695833

[30] Beutler B Rietschel ET. Innate immune sensing and its roots: the story of endotoxin. Nat Rev Immunol (2003) 3(2):169–76. 10.1038/nri1004 12563300

[31] Han J Ulevitch RJ. Limiting inflammatory responses during activation of innate immunity. Nat Immunol (2005) 6(12):1198–205. 10.1038/ni1274 16369559

[32] Hayden MS Ghosh S Signaling to NF-kappaB. Genes Dev (2004) 18(18):2195–224. 10.1101/gad.1228704 15371334

[33] Chakrawarti L Agrawal R Dang S Gupta S Gabrani R Therapeutic effects of EGCG: a patent review. Expert Opin Ther Pat (2016) 26(8):907–16. 10.1080/13543776.2016.1203419 27338088

[34] Lagha AB Grenier D Tea polyphenols inhibit the activation of NF-kappaB and the secretion of cytokines and matrix metalloproteinases by macrophages stimulated with *Fusobacterium nucleatum*. Sci Rep (2016) 6:34520 10.1038/srep34520 27694921 PMC5046134

[35] Wu M Liu D Zeng R Xian T Lu Y Zeng G Epigallocatechin-3-gallate inhibits adipogenesis through down-regulation of PPARgamma and FAS expression mediated by PI3K-AKT signaling in 3T3-L1 cells. Eur J Pharmacol (2016) 795:134–42. 10.1016/j.ejphar.2016.12.006 27940057

[36] Yu L Yu H Li X Jin C Zhao Y Xu S P38 MAPK/miR-1 are involved in the protective effect of EGCG in high glucose-induced Cx43 downregulation in neonatal rat cardiomyocytes. Cell Biol Int (2016) 40(8):934–42. 10.1002/cbin.10637 27306406

[37] Kim HS Kim MH Jeong M Hwang YS Lim SH Shin BA EGCG blocks tumor promoter-induced MMP-9 expression via suppression of MAPK and AP-1 activation in human gastric AGS cells. Anticancer Res (2004) 24(2B):747–53. 15161022

[38] Andersson ER Sandberg R Lendahl U. Notch signaling: simplicity in design, versatility in function. Development (2011) 138(17):3593–612. 10.1242/dev.063610 21828089

