# Supplementary material for: (−)-Epigallocatechin Gallate Targets Notch to Attenuate the Inflammatory Response in the Immediate Early Stage in Human Macrophages
Source: Front Immunol. 2017 Apr 10;8:433. doi: 10.3389/fimmu.2017.00433 (PMC5385462; doi:10.3389/fimmu.2017.00433)
Supplement: Supplementary file 1 [file Data_Sheet_1.DOCX]

**Supplementary data**

**Supplementary Fig. 1** THP-1 (left) and THP-1 derived macrophages (right). Scale bar = 50 μm.

**Supplementary Fig. 2** THP-1 cells were treated with PMA (10 ng/mL) from 0 h to 48 h. *cd14* mRNA levels were measured by real-time PCR.

**Supplementary Fig. 3** THP-1-derived macrophages were preincubated with 67LR antibody (5 μg/mL) 1 h and treated with EGCG (50 μg/mL) for 30 min followed by exposure to LPS (200 EU/mL) for 3 h. Cell lysates were probed with an antibody against pNF-κB (2A) and MAPK (p38, p42/44 and JNK) (2B).

**Supplementary Fig. 4** Major inflammatory cytokines release 3 hours after LPS treatment on macrophages derived from THP-1, THP-1/siNotch1 or THP-1/siNotch2.

**Supplementary Fig. 5** THP-1-derived macrophages were treated with EGCG (50 μg/mL) from 6 h to 18 h. Cell lysates were probed with antibodies against Notch1 or Notch2.

**Supplementary Fig. 1**


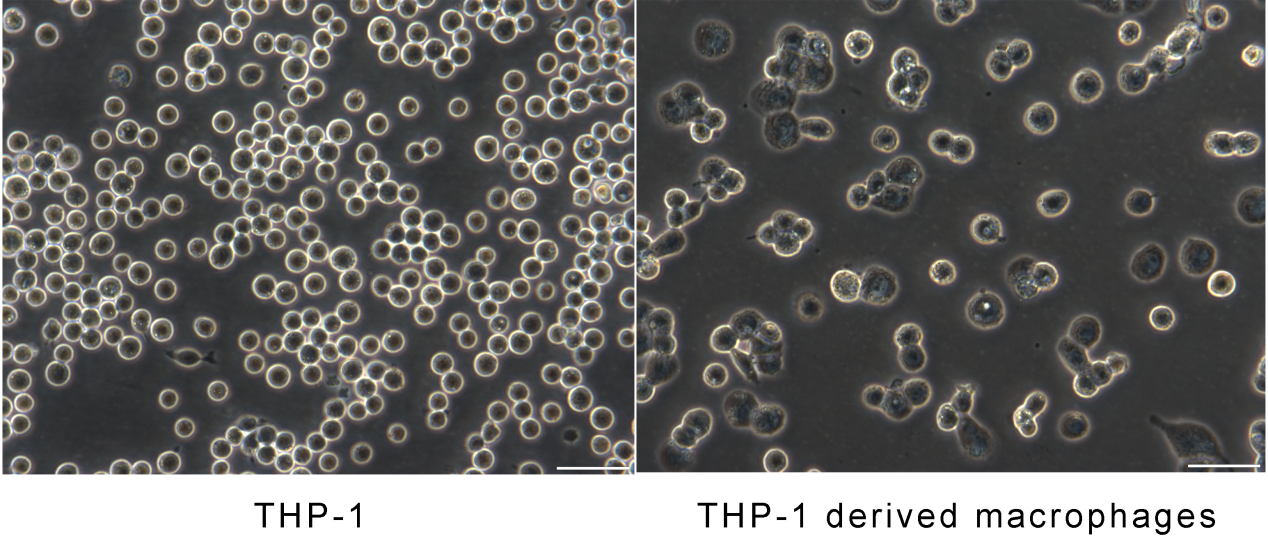


**Supplementary Fig. 2**





**Supplementary Fig. 3**

**A**


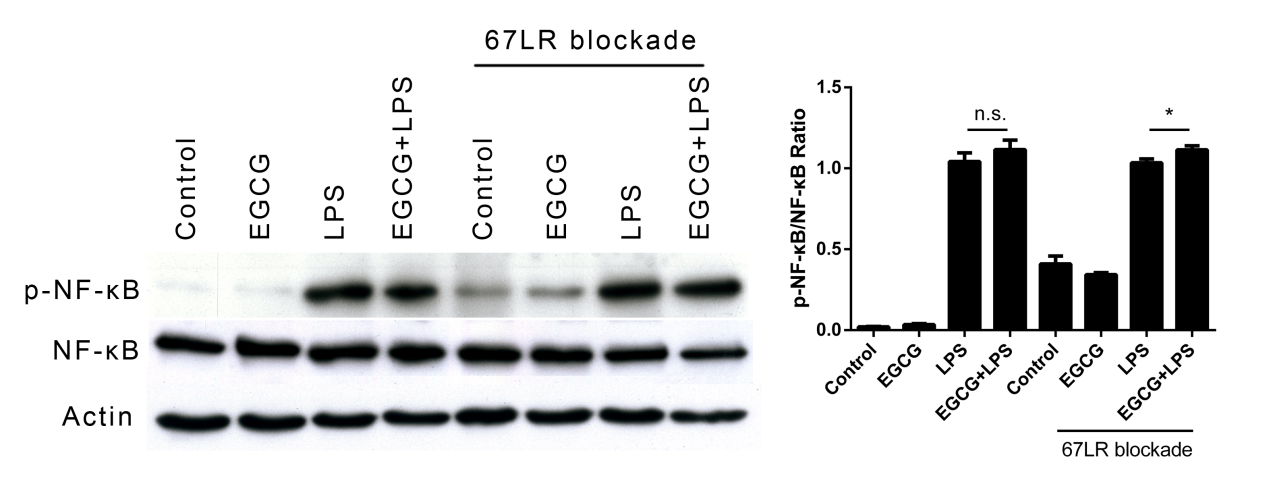


**B**

**
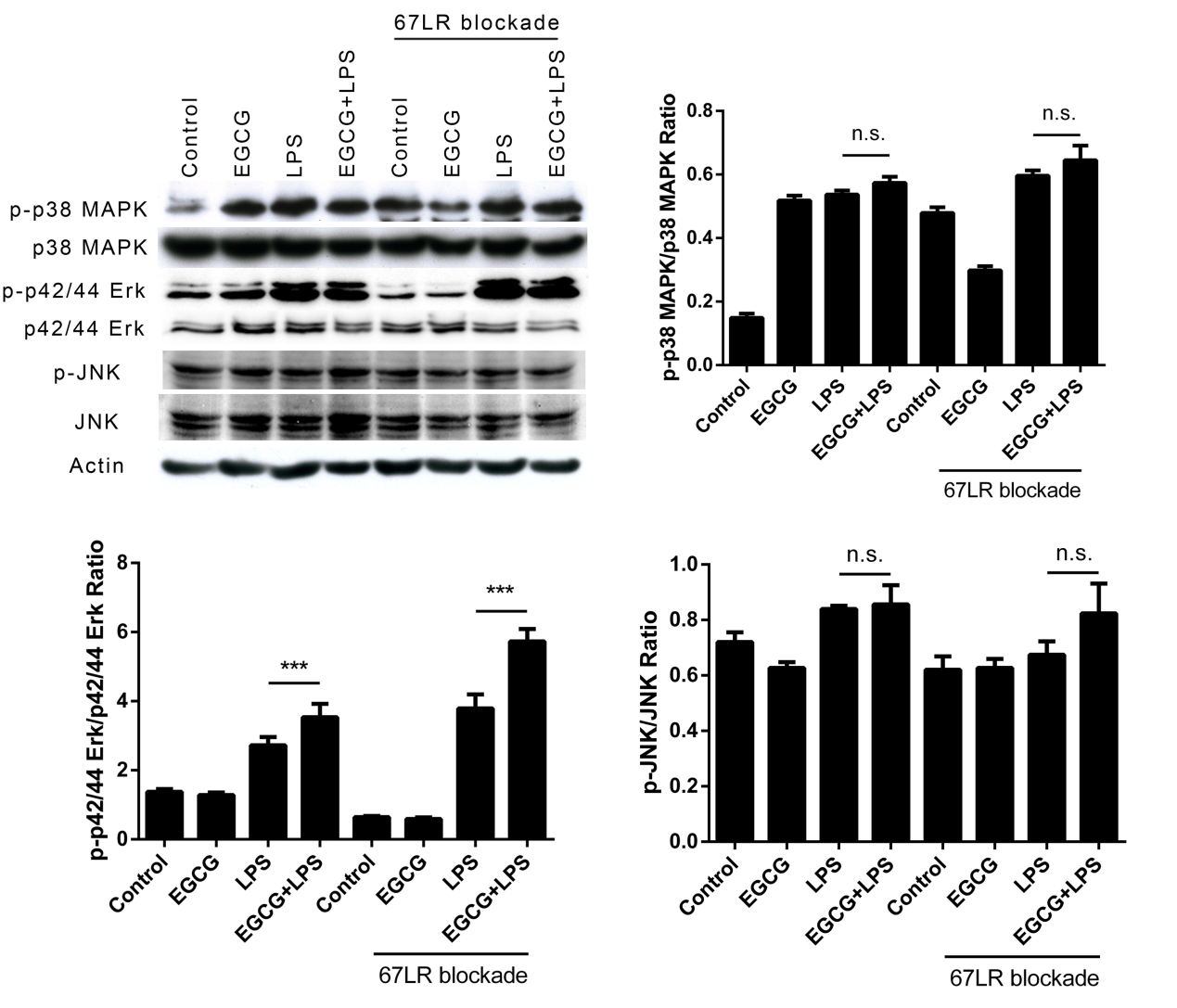
**

**Supplementary Fig. 4**

**

**

**Supplementary Fig. 5**

**
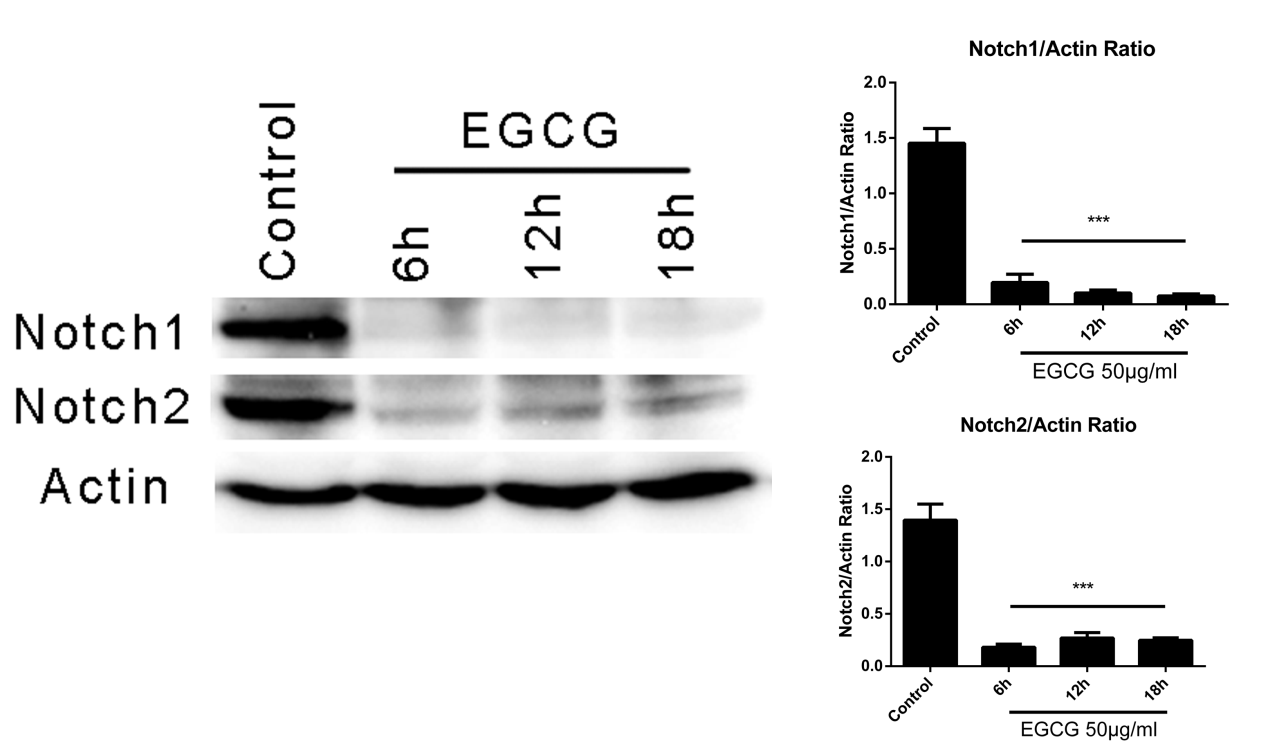
**
