# Supplementary material for: (−)-Epigallocatechin Gallate Targets Notch to Attenuate the Inflammatory Response in the Immediate Early Stage in Human Macrophages
Source: Front Immunol. 2017 Apr 10;8:433. doi: 10.3389/fimmu.2017.00433 (PMC5385462; doi:10.3389/fimmu.2017.00433)
Supplement: Supplementary file 2 [file Table_1.DOCX]

Supplement Table 1. Primers for the target genes in quantitative RT-PCR.

| Genes | Accession | Forward primer | Reverse primer |
| --- | --- | --- | --- |
| *actb* | NM_001101 | CTACCTCATGAAGATCCTCACCGA | TTCTCCTTAATGTCACGCACGATT |
| *ccl3* | NM_002983 | CTCTCTGCAACCAGTTCTCT | TGCTCGTCTCAAAGTAGTCA |
| *ccl4* | NM_002984 | CCTGCTGCTTTTCTTACAC | TTGCTTCTTTTGGTTTGG |
| *ccl5* | NM_001278736 | CCAACCCAGAGAAGAAATG | GGACAAGAGCAAGCAGAA |
| *il1b（il-1 beta）* | NM_000576 | ACCACCACTACAGCAAGG | AAAGATGAAGGGAAAGAAGG |
| *il6* | NM_000600 | CTGGTCTTTTGGAGTTTGAG | GGTCAGGGGTGGTTATTG |
| *cxcl8(il8)* | NM_000584 | GCATAAAGACATACTCCAAACC | AACTTCTCCACAACCCTCT |
| *il10* | NM_000572 | GGAGAACCTGAAGACCCT | GCTTTGTAGATGCCTTTC |
| *ccl2(mcp1)* | NM_002982 | AGAATCACCAGCAGCAAG | GTCTTCGGAGTTTGGGTT |
| *timp2* | NM_003255 | AGCACCACCCAGAAGAAG | GACCCAGTCCATCCAGAG |
| *tnf（tnf alpha）* | NM_000594 | CAACCTCCTCTCTGCCAT | GAAGACCCCTCCCAGATA |
| *bhlhe40* | NM_003670 | TAAGCAAGAGTCCGAAGAAC | GTAGAAGGGCAGGCAGAA |
| *ddit4* | NM_019058 | TGACCCTGAGGATGAACACTT | CTTTGCCCACCTGGCTTAC |
| *elmo1* | NM_014800 | GTATCTCTCTCCTCACGCA | CACCGAAAATGTATCCCA |
| *hes1* | [NM_005524](https://www.ncbi.nlm.nih.gov/nuccore/NM_005524.3) | AGGTGCTTCACTGTCATTTCC | AACACTGATTTTGGATGCTCTG |
| *hey1* | [NM_001040708](https://www.ncbi.nlm.nih.gov/nuccore/NM_001040708.1) | CCCAAACTCCGATAGTCCATA | CTGAGCTGAGAAGGCTGGTAC |
| *hk2* | NM_000189 | CATCTGCTTGCCTACTTCTT | CCCTTTCTCCATCTCCTT |
| *p4ha1* | NM_000917 | GCTATGAAAATCCTGTGGTG | CAAAGTCAAAATGGGGTTC |
| *pfkfb3* | NM_001282630 | ACCTACCCTGAGGAGTATGCG | ATCCAGGAAGTAGGCAAGCAG |
| *rhou* | NM_021205 | CCTGCTCTGCTTCAGTGTCGT | CTGGCTTTTCTTTGCATTTGTC |
| *cd14* | [NM_000591](https://www.ncbi.nlm.nih.gov/nuccore/NM_000591.3) | CGAGGACCTAAAGATAACCGGC | GTTGCAGCTGAGATCGAGCAC |
