# Supplementary material for: (−)-Epigallocatechin Gallate Targets Notch to Attenuate the Inflammatory Response in the Immediate Early Stage in Human Macrophages
Source: Front Immunol. 2017 Apr 10;8:433. doi: 10.3389/fimmu.2017.00433 (PMC5385462; doi:10.3389/fimmu.2017.00433)
Supplement: Supplementary file 3 [file Table_2.DOCX]

Supplementary Table 2 Relative expression of inflammatory factors in THP-1-derived macrophages

|  | Control | EGCG | LPS | EGCG+LPS | Significance  (LPS *vs.* EGCG+LPS) |
| --- | --- | --- | --- | --- | --- |
| Eotaxin | 2485.33±271.53 | 2183.89±269.54 | 4599.59±687.73 | 3230.36±701.45 | n.s. |
| Eotaxin-2 | 1845.33±271.53 | 1087.97±741.24 | 3566.20±257.90 | 1322.67±269.79 | * |
| G-CSF | 245.33±0.00 | 0.00±0.00 | 405.25±257.90 | 0.00±0.00 | n.s |
| GM-CSF | 0.00±0.00 | 0.00±0.00 | 415.38±587.44 | 0.00±0.00 | n.s |
| ICAM-1 | 1589.33±633.57 | 91.33±129.15 | 3140.69±171.93 | 101.74±53.96 | ** |
| IFN-gamma | 1269.33±1086.12 | 43.68±61.77 | 1134.70±85.97 | 0.00±0.00 | ** |
| I-309 | 2357.33±1538.66 | 2231.54±67.39 | 2532.81±2063.20 | 1367.18±1933.48 | n.s |
| IL-1a | 1589.33±814.59 | 91.33 ±129.15 | 2168.09±0.00 | 0.00±0.00 | ** |
| IL-1beta | 2421.33±181.02 | 659.14±134.77 | 11650.93±343.87 | 1284.51±431.66 | ** |
| IL-2 | 309.33±90.51 | 0.00±0.00 | 526.82±257.90 | 0.00±0.00 | n.s |
| IL-3 | 629.33±181.02 | 43.68 ±61.77 | 1924.94±171.93 | 0.00±0.00 | ** |
| IL-4 | 1077.33±452.55 | 420.89±67.39 | 1499.42±85.97 | 216.21±107.92 | ** |
| IL-6 | 1973.33±90.51 | 0.00±0.00 | 12198.02±773.70 | 0.00±0.00 | ** |
| IL-6 sR | 1909.33±724.08 | 0.00±0.00 | 7152.66±687.73 | 0.00±0.00 | ** |
| IL-7 | 437.33±271.53 | 0.00±0.00 | 466.04±343.87 | 0.00±0.00 | n.s |
| IL8 | 31605.33±1086.12 | 4995.14±202.16 | 52804.06±85.97 | 11013.74±2859.76 | ** |
| IL-10 | 2677.33±362.04 | 468.54±134.77 | 8246.84±859.66 | 1551.59±593.53 | * |
| IL-11 | 58.67±82.97 | 0.00±0.00 | 111.44±157.61 | 0.00±0.00 | n.s. |
| IL12-p40 | 885.33±181.02 | 135.00±67.39 | 1681.79±1031.60 | 0.00±0.00 | n.s. |
| IL12-p70 | 437.33±271.53 | 0.00±0.00 | 1681.79±171.93 | 0.00±0.00 | ** |
| IL-13 | 693.33±271.53 | 138.97±196.54 | 1803.36±343.87 | 0.00±0.00 | * |
| IL-15 | 1141.33±362.04 | 659.14±269.54 | 6727.15±1977.23 | 979.28±0.00 | n.s. |
| IL-16 | 885.33±543.06 | 0.00±0.00 | 2107.30±429.83 | 0.00±0.00 | * |
| IL17 | 245.33±0.00 | 0.00±0.00 | 344.46±171.93 | 0.00±0.00 | n.s. |
| IP-10 | 3509.33±452.55 | 3565.69±202.16 | 12076.45±8166.82 | 3764.51±53.96 | n.s. |
| MCP-1 | 15029.33±452.55 | 5090.44±336.93 | 23930.01±687.73 | 4336.82±107.92 | *** |
| MCP-2 | 949.33±452.55 | 373.25±134.77 | 2168.09±515.80 | 101.74±53.96 | * |
| M-CSF | 1653.33±362.04 | 91.33±129.15 | 2350.45±773.70 | 146.26±206.84 | n.s. |
| MIG | 122.67±173.48 | 0.00±0.00 | 233.02±329.54 | 0.00±0.00 | n.s. |
| CCL3 | 2741.33±90.51 | 7329.92±269.54 | 24233.94±5587.82 | 8800.82±917.28 | n.s. |
| CCL4 | 17525.33±5249.56 | 13190.68±2897.56 | 34385.45±1031.60 | 11776.82±1133.11 | ** |
| MIP-1-delta | 821.33±90.51 | 186.62±263.92 | 1134.70±601.77 | 108.10±152.88 | n.s. |
| CCL5 | 7669.33±1086.12 | 992.68±202.16 | 21133.78±2063.20 | 1589.74±107.92 | ** |
| TGF-beta 1 | 1589.33±271.53 | 1230.92±0.00 | 4599.59±687.73 | 1666.05±0.00 | * |
| TNF-alpha | 373.33±0.00 | 611.49±202.16 | 27759.62±2149.16 | 2925.13±485.62 | ** |
| TNF-beta | 1909.33±181.02 | 2374.48±673.85 | 5632.97±601.77 | 2429.13±971.24 | n.s. |
| sTNF-RI | 2229.33±452.55 | 563.84±0.00 | 5693.76±687.73 | 712.21±161.87 | ** |
| sTNF RII | 2293.33±181.02 | 2183.89±269.54 | 6362.42±945.63 | 3039.59±107.92 | * |
| PDGF-BB | 1845.33±633.57 | 2755.67±0.00 | 3809.35±85.97 | 2543.59±53.96 | ** |
| TIMP-2 | 8181.33±181.02 | 1373.86±336.93 | 14021.65±1633.36 | 826.67±0.00 | ** |
