# Supplementary material for: (−)-Epigallocatechin Gallate Targets Notch to Attenuate the Inflammatory Response in the Immediate Early Stage in Human Macrophages
Source: Front Immunol. 2017 Apr 10;8:433. doi: 10.3389/fimmu.2017.00433 (PMC5385462; doi:10.3389/fimmu.2017.00433)
Supplement: Supplementary file 4 [file Table_3.DOCX]

Supplement Table 3 Relative expression of inflammatory factors in THP-1-derived macrophages with 67LR blockade

|  | Control | EGCG | LPS | EGCG+LPS | Significance  (LPS *vs.* EGCG+LPS) |
| --- | --- | --- | --- | --- | --- |
| Eotaxin | 1156.00±181.02 | 514.00 ±0.00 | 2184.00 ±181.02 | 514.00 ±0.00 | ** |
| Eotaxin-2 | 771.00±363.45 | 514.00 ±0.00 | 1285.00 ±1.41 | 0.00 ±0.00 | *** |
| G-CSF | 0.00 ±0.00 | 0.00 ±0.00 | 0.00 ±0.00 | 0.00 ±0.00 |  |
| GM-CSF | 0.00 ±0.00 | 206.26 ±291.70 | 0.00 ±0.00 | 0.00 ±0.00 |  |
| ICAM-1 | 642.00 ±181.02 | 0.00 ±0.00 | 1231.46± 157.65 | 0.00 ±0.00 | **. |
| IFN-gamma | 257.00 ±363.45 | 0.00 ±0.00 | 447.65 ±0.00 | 0.00 ±0.00 | *** |
| I-309 | 257.00 ±363.45 | 206.26 ±291.70 | 559.12 ±157.65 | 0.00 ±0.00 | *. |
| IL-1a | 0.00 ±0.00 | 0.00 ±0.00 | 447.65 ±0.00 | 0.00 ±0.00 | *** |
| IL-1beta | 643.00 ±182.43 | 0.00 ±0.00 | 1454.42 ±157.65 | 103.92 ±146.97 | * |
| IL-2 | 257.00 ±363.45 | 0.00 ±0.00 | 448.52 ±316.53 | 103.92 ±146.97 | n.s. |
| IL-3 | 514.00 ±0.00 | 0.00 ±0.00 | 447.65 ±0.00 | 207.04 ±292.80 | n.s. |
| IL-4 | 0.00 ±0.00 | 0.00 ±0.00 | 223.82 ±1.23 | 0.00 ±0.00 | ***. |
| IL-6 | 0.00 ±0.00 | 0.00 ±0.00 | 671.47 ±1.23 | 0.00 ±0.00 | *** |
| IL-6 sR | 1542.00± 0.00 | 0.00 ±0.00 | 3357.35 ±316.53 | 0.00 ±0.00 | ** |
| IL-7 | 257.00 ±363.45 | 0.00 ±0.00 | 0.00 ±0.00 | 0.00 ±0.00 |  |
| IL8 | 49344.00 ±363.45 | 3609.17± 146.42 | 43421.75± 949.60 | 5900.19 ±439.76 | *** |
| IL-10 | 1670.00 ±181.02 | 0.00 ±0.00 | 1902.06 ±475.42 | 0.00 ±0.00 | * |
| IL-11 | 0.00 ±0.00 | 0.00 ±0.00 | 112.35 ±158.88 | 0.00 ±0.00 | n.s. |
| IL12-p40 | 257.00 ±363.45 | 0.00 ±0.00 | 782.95 ±158.88 | 0.00 ±0.00 | * |
| IL12-p70 | 514.00 ±0.00 | 0.00± 0.00 | 447.65 ±0.00 | 0.00 ±0.00 | *** |
| IL-13 | 0.00 ±0.00 | 0.00 ±0.00 | 336.17 ±157.65 | 0.00 ±0.00 | n.s. |
| IL-15 | 899.00 ±182.43 | 0.00 ±0.00 | 1566.76 ±316.53 | 0.00 ±0.00 | * |
| IL-16 | 514.00 ±0.00 | 0.00 ±0.00 | 671.47 ±1.23 | 0.00 ±0.00 | *** |
| IL17 | 0.00 ±0.00 | 0.00 ±0.00 | 224.69 ±0.00 | 0.00 ±0.00 | *** |
| IP-10 | 2184.00± 181.02 | 1856.35 ±291.70 | 2797.36 ±475.42 | 828.96 ±292.80 | * |
| MCP-1 | 1284.00 ±0.00 | 206.26 ±291.70 | 1678.24 ±158.88 | 0.00 ±0.00 | ** |
| MCP-2 | 257.00 ±363.45 | 0.00 ±0.00 | 447.65 ±0.00 | 0.00 ±0.00 | *** |
| M-CSF | 771.00 ±363.45 | 0.00 ±0.00 | 1231.46 ±475.42 | 0.00 ±0.00 | n.s. |
| MIG | 257.00 ±363.45 | 0.00 ±0.00 | 111.48 ±157.65 | 207.04 ±292.80 | n.s. |
| CCL3 | 2312.00 ±0.00 | 3506.44 ±291.70 | 7721.47 ±1423.79 | 4450.92 ±1024.22 | n.s. |
| CCL4 | 9638.00 ±181.02 | 7631.67 ±291.70 | 13653.23 ±949.60 | 9109.69 ±1171.19 | n.s. |
| MIP-1-delta | 128.00 ±181.02 | 0.00 ±0.00 | 671.47 ±316.53 | 0.00 ±0.00 | n.s. |
| CCL5 | 12207.00 ±1998.28 | 0.00 ±0.00 | 22102.79 ±2452.22 | 0.00 ±0.00 | ** |
| TGF-beta 1 | 1156.00 ±181.02 | 412.52 ±0.00 | 782.95 ±474.19 | 207.04 ±292.80 | n.s. |
| TNF-alpha | 514.00 ±0.00 | 0.00 ±0.00 | 20143.68 ±2213.89 | 414.08 ±0.00 | ** |
| TNF-beta | 1156.00 ±181.02 | 103.53 ±146.42 | 3021.18 ±474.19 | 414.08 ±0.00 | * |
| sTNF-RI | 899.00 ±182.43 | 0.00 ±0.000.00 | 1007.64 ±158.88 | 0.00 ±0.00 | * |
| sTNF RII | 1413.00 ±182.43 | 206.26 ±291.70 | 2125.89 ±158.88 | 0.00 ±0.00 | ** |
| PDGF-BB | 1156.00±181.02 | 514.00 ±0.00 | 2184.00 ±181.02 | 514.00 ±0.00 | * |
| TIMP-2 | 771.00±363.45 | 514.00 ±0.00 | 1285.00 ±1.41 | 0.00 ±0.00 | ** |
