# Supplementary material for: (−)-Epigallocatechin Gallate Targets Notch to Attenuate the Inflammatory Response in the Immediate Early Stage in Human Macrophages
Source: Front Immunol. 2017 Apr 10;8:433. doi: 10.3389/fimmu.2017.00433 (PMC5385462; doi:10.3389/fimmu.2017.00433)
Supplement: Supplementary file 5 [file Table_4.DOCX]

Supplementary Table 4 Relative expression of inflammatory factors in THP-1/siNotch1 derived macrophages

|  | Control | EGCG | LPS | EGCG+LPS | Significance  (LPS *vs.* EGCG+LPS) |
| --- | --- | --- | --- | --- | --- |
| Eotaxin | 2430.05±640.72 | 6412.51±420.98 | 2453.89±68.05 | 6635.01±175.21 | **. |
| Eotaxin-2 | 1812.24±815.47 | 3696.18±52.62 | 3368.09±136.09 | 1638.10±467.22 | * |
| G-CSF | 0.00±0.00 | 12.40±0.00 | 192.46±0.00 | 34.41±48.67 | * |
| GM-CSF | 0.00±0.00 | 124.03±52.62 | 240.58±68.05 | 0.00±0.00 | * |
| ICAM-1 | 1317.99±232.99 | 1314.75±684.10 | 1924.62±1497.01 | 936.06±876.04 | n.s. |
| IFN-gamma | 741.37±232.99 | 1389.17±473.60 | 625.50±204.14 | 199.60±282.28 | n.s. |
| I-309 | 1153.24±582.48 | 2989.19±1683.93 | 866.08±816.55 | 1142.54±817.63 | n.s |
| IL-1a | 1894.61±116.50 | 3138.03±420.98 | 1106.66±476.32 | 646.98±467.22 | n.s. |
| IL-1beta | 453.06±58.25 | 1240.33±157.87 | 8275.88±136.09 | 7667.42±233.61 | n.s. |
| IL-2 | 205.94±291.24 | 310.08±210.49 | 288.69±0.00 | 481.80±116.80 | n.s. |
| IL-3 | 82.37±116.50 | 1538.01±263.11 | 721.73±204.14 | 523.09±58.40 | n.s. |
| IL-4 | 41.19±58.25 | 1761.27±157.87 | 673.62±136.09 | 936.06±175.21 | n.s. |
| IL-6 | 0.00±0.00 | 161.24±0.00 | 96.23±136.09 | 0.00±0.00 | n.s. |
| IL-6 sR | 1688.68±58.25 | 3621.76±684.10 | 5244.60±1292.87 | 3992.02±408.82 | n.s. |
| IL-7 | 0.00±0.00 | 124.03±52.62 | 192.46±0.00 | 34.41±48.67 | * |
| IL8 | 14868.61±990.21 | 11547.47±315.74 | 27907.04±5307.57 | 22410.35±3912.96 | n.s. |
| IL-10 | 1894.61±349.49 | 3696.18±473.60 | 9959.93±884.59 | 3702.94±0.00 | ** |
| IL-11 | 205.94±58.25 | 198.45±157.87 | 384.92±544.37 | 34.41±48.67 | n.s. |
| IL12-p40 | 782.56±407.73 | 942.65±789.34 | 288.69±408.27 | 275.31±175.21 | n.s. |
| IL12-p70 | 329.50±349.49 | 1054.28±526.23 | 769.85±544.37 | 240.90±340.68 | n.s. |
| IL-13 | 123.56±58.25 | 793.81±52.62 | 673.62±136.09 | 977.36±0.00 | n.s. |
| IL-15 | 1235.62±698.97 | 2319.42±210.49 | 2742.59±1020.69 | 2174.96±58.40 | n.s. |
| IL-16 | 370.69±58.25 | 1351.96±105.25 | 1395.35±204.14 | 1142.54±350.41 | n.s. |
| IL17 | 123.56±174.74 | 310.08±210.49 | 144.35±68.05 | 158.30±223.88 | n.s. |
| IP-10 | 2018.18±58.25 | 4477.59±420.98 | 7169.22±748.50 | 2959.60±1518.46 | n.s. |
| MCP-1 | 2224.11±349.49 | 2356.63±263.11 | 11451.51±1088.73 | 11136.35±5723.43 | n.s. |
| MCP-2 | 535.43±58.25 | 1314.75±157.87 | 1154.77±0.00 | 936.06±58.40 | * |
| M-CSF | 906.12±465.98 | 1835.69±52.62 | 2405.78±272.18 | 936.06±408.82 | n.s. |
| MIG | 0.00±0.00 | 272.87±52.62 | 96.23±136.09 | 0.00±0.00 | n.s. |
| CCL3 | 2718.36±1397.94 | 3621.76±52.62 | 16070.60±680.46 | 14976.94±58.40 | n.s. |
| CCL4 | 10008.51±58.25 | 11770.73±841.96 | 19246.23±272.18 | 16504.92±4438.58 | n.s. |
| MIP-1-delta | 123.56±174.74 | 496.13±52.62 | 240.58±68.05 | 275.31±58.40 | n.s. |
| CCL5 | 3871.60±349.49 | 2654.31±157.87 | 7361.68±68.05 | 3124.79±116.80 | *** |
| TGF-beta 1 | 1194.43±407.73 | 2096.16±105.25 | 2261.43±748.50 | 1596.81±58.40 | n.s. |
| TNF-alpha | 453.06±174.74 | 1798.48±0.00 | 17129.15±816.55 | 15513.80±116.80 | n.s. |
| TNF-beta | 1606.30±873.71 | 7305.54±420.98 | 5196.48±544.37 | 3083.49±408.82 | * |
| sTNF-RI | 823.75±116.50 | 2096.16±736.72 | 3416.21±68.05 | 2381.44±1168.05 | n.s. |
| sTNF RII | 1400.37±232.99 | 2840.36±631.47 | 2983.17±408.27 | 3166.08±175.21 | n.s. |
| PDGF-BB | 1317.99±1048.46 | 2282.21±157.87 | 2502.01±136.09 | 2133.66±116.80 | n.s. |
| TIMP-2 | 8814.08±0.00 | 6003.20±578.85 | 11595.86±68.05 | 6552.41±408.82 | ** |
