# Supplementary material for: (−)-Epigallocatechin Gallate Targets Notch to Attenuate the Inflammatory Response in the Immediate Early Stage in Human Macrophages
Source: Front Immunol. 2017 Apr 10;8:433. doi: 10.3389/fimmu.2017.00433 (PMC5385462; doi:10.3389/fimmu.2017.00433)
Supplement: Supplementary file 6 [file Table_5.DOCX]

Supplementary Table 5 Relative expression of inflammatory factors in THP-1/siNotch2 derived macrophages

|  | Control | EGCG | LPS | EGCG+LPS | Significance  (LPS *vs.* EGCG+LPS) |
| --- | --- | --- | --- | --- | --- |
| Eotaxin | 7880.67±241.36 | 5396.68±1695.67 | 7616.47±396.69 | 7908.26±109.03 | n.s. |
| Eotaxin-2 | 3769.67±242.77 | 2561.81±549.98 | 3786.09±314.11 | 3768.37±457.27 | n.s. |
| G-CSF | 556.67±60.34 | 361.40±20.17 | 661.91±392.31 | 562.62±90.47 | n.s |
| GM-CSF | 556.67±60.34 | 42.89±60.65 | 1238.80±426.35 | 875.91±533.54 | n.s. |
| ICAM-1 | 1970.67±241.36 | 1348.66±423.20 | 2840.30±204.03 | 2400.77±149.33 | n.s. |
| IFN-gamma | 1584.67±60.34 | 958.64±128.38 | 1751.61±298.87 | 1937.18±81.19 | n.s. |
| I-309 | 1969.67±122.09 | 1218.99±239.82 | 1751.61±298.87 | 1937.18±81.19 | n.s. |
| IL-1a | 1585.67±423.79 | 1462.55±265.00 | 3129.25±204.60 | 3013.48±168.95 | n.s. |
| IL-1beta | 685.67±242.77 | 1088.31±55.00 | 16334.03±1144.19 | 11442.40±654.53 | * |
| IL-2 | 299.67±303.11 | 248.53±139.46 | 711.53±764.98 | 412.33±303.02 | n.s. |
| IL-3 | 941.67±484.13 | 846.64±30.01 | 1478.68±321.53 | 1331.37±330.77 | n.s. |
| IL-4 | 1198.67±120.68 | 604.09±6.25 | 1495.70±63.03 | 1482.89±119.94 | n.s. |
| IL-6 | 298.67±60.34 | 173.57±245.47 | 1095.89±221.42 | 562.62±90.47 | n.s. |
| IL-6 sR | 5568.67±120.68 | 3905.51±858.91 | 19554.61±268.79 | 15845.83±1586.99 | n.s. |
| IL-7 | 1070.67±60.34 | 43.90±62.08 | 1223.34±41.17 | 862.04±332.98 | n.s. |
| IL8 | 19445.67±242.77 | 8514.26±1335.51 | 32468.40±801.08 | 37133.69±1120.99 | * |
| IL-10 | 4925.67±303.11 | 2076.58±499.80 | 14328.02±1329.42 | 7138.34±347.67 | * |
| IL-11 | 427.67±242.77 | 42.89±60.65 | 805.95±185.80 | 712.91±122.08 | n.s. |
| IL12-p40 | 1840.67±60.34 | 1330.99±81.42 | 1896.64±93.77 | 2250.48±361.88 | n.s. |
| IL12-p70 | 1841.67±58.93 | 1218.99±239.82 | 2024.09±274.02 | 1937.18±81.19 | n.s. |
| IL-13 | 813.67±423.79 | 492.09±164.65 | 1205.76±344.18 | 725.62±746.08 | n.s. |
| IL-15 | 4410.67±60.34 | 5928.57±1495.04 | 8945.62±255.55 | 7915.22±325.92 | n.s. |
| IL-16 | 1327.67±61.75 | 1106.99±398.21 | 2585.52±159.47 | 1788.05±129.71 | * |
| IL17 | 556.67±60.34 | 42.89±60.65 | 534.58±209.06 | 712.91±122.08 | n.s. |
| IP-10 | 11350.67±1393.47 | 11910.88±1686.76 | 13899.86±1320.81 | 14635.49±757.99 | n.s. |
| MCP-1 | 4796.67±485.55 | 3532.28±650.34 | 8290.90±263.34 | 8527.94±345.53 | n.s. |
| MCP-2 | 1455.67±122.09 | 1106.99±398.21 | 1768.19±87.89 | 1788.05±129.71 | n.s. |
| M-CSF | 2291.17±29.46 | 1266.16±10.27 | 2697.39±408.95 | 2386.90±717.18 | n.s. |
| MIG | 428.67±241.36 | 43.90±62.08 | 278.68±152.84 | 313.30±443.07 | n.s. |
| CCL3 | 3511.67±122.09 | 2918.25±415.54 | 16429.57±195.00 | 21237.74±535.16 | ** |
| CCL4 | 11606.67±666.57 | 8532.95±1678.71 | 20578.24±1178.83 | 26411.78±1461.80 | * |
| MIP-1-delta | 298.67±60.34 | 304.26±430.28 | 1205.76±344.18 | 1012.33±545.52 | n.s. |
| CCL5 | 2869.67±303.11 | 3401.59±465.52 | 10646.23±1425.67 | 7003.08±729.74 | n.s. |
| TGF-beta 1 | 2355.67±423.79 | 3176.72±783.54 | 5164.73±187.95 | 4851.64±227.81 | n.s. |
| TNF-alpha | 942.67±241.36 | 604.09±6.25 | 17407.27±853.86 | 23553.41±1694.43 | * |
| TNF-beta | 4924.67±666.57 | 6461.34±1293.18 | 7617.60±398.28 | 8378.81±556.43 | n.s. |
| sTNF-RI | 1841.67±303.11 | 2076.58±499.80 | 3691.67±636.67 | 4238.92±208.19 | n.s. |
| sTNF RII | 4025.67±122.09 | 3532.28±650.34 | 5965.48±127.24 | 7138.34±347.67 | * |
| PDGF-BB | 5438.67±60.34 | 4615.61±591.07 | 5709.57±234.67 | 7294.38±127.00 | * |
| TIMP-2 | 7880.67±241.36 | 5396.68±1695.67 | 7616.47±396.69 | 7908.26±109.03 | n.s. |
